# Amelioration of inflammatory myopathies by glucagon‐like peptide‐1 receptor agonist via suppressing muscle fibre necroptosis

**DOI:** 10.1002/jcsm.13025

**Published:** 2022-06-30

**Authors:** Mari Kamiya, Fumitaka Mizoguchi, Shinsuke Yasuda

**Affiliations:** ^1^ Department of Rheumatology, Graduate School of Medical and Dental Sciences Tokyo Medical and Dental University (TMDU) Tokyo Japan

**Keywords:** Inflammatory myopathies, Polymyositis, GLP‐1R agonist, Necroptosis, Oxidative stress

## Abstract

**Background:**

As glucocorticoids induce muscle atrophy during the treatment course of polymyositis (PM), novel therapeutic strategy is awaited that suppresses muscle inflammation but retains muscle strength. We recently found that injured muscle fibres in PM undergo FASLG‐mediated necroptosis, a form of regulated cell death accompanied by release of pro‐inflammatory mediators, contributes to accelerate muscle inflammation and muscle weakness. Glucagon‐like peptide‐1 receptor (GLP‐1R) agonists have pleiotropic actions including anti‐inflammatory effects, prevention of muscle atrophy, and inhibition of cell death, in addition to anti‐diabetic effect. We aimed in this study to examine the role of GLP‐1R in PM and the effect of a GLP‐1R agonist on *in vivo* and *in vitro* models of PM.

**Methods:**

Muscle specimens of PM patients and a murine model of PM, C protein‐induced myositis (CIM), were examined for the expression of GLP‐1R. The effect of PF1801, a GLP‐1R agonist, on CIM was evaluated in monotherapy or in combination with prednisolone (PSL). As an *in vitro* model of PM, C2C12‐derived myotubes were treated with FASLG to induce necroptosis. The effect of PF1801 on this model was analysed.

**Results:**

GLP‐1R was expressed on the inflamed muscle fibres of PM and CIM. The treatment of CIM with PF1801 in monotherapy (PF) or in combination with PSL (PF + PSL) suppressed CIM‐induced muscle weakness (grip strength, mean ± SD (g); PF 227 ± 6.0 (*P* < 0.01), PF + PSL 224 ± 8.5 (*P* < 0.01), Vehicle 162 ± 6.0) and decrease in cross‐sectional area of muscle fibres (mean ± SD (μm^2^); PF 1896 ± 144 (*P* < 0.05), PF + PSL 2018 ± 445 (*P* < 0.01), Vehicle 1349 ± 199) as well as the severity of histological inflammation scores (median, interquartile range; PF 0.0, 0.0–0.5 (*P* < 0.05), PF + PSL 0.0, 0.0–0.0 (*P* < 0.01), Vehicle 1.9, 1.3–3.3). PF1801 decreased the levels of inflammatory mediators such as TNFα, IL‐6, and HMGB1 in the serum of CIM. PF1801 inhibited necroptosis of the myotubes in an AMP‐activated protein kinase (AMPK)‐dependent manner. PF1801 activated AMPK and decreased the expression of PGAM5, a mitochondrial protein, which was crucial for necroptosis of the myotubes. PF1801 promoted the degradation of PGAM5 through ubiquitin‐proteasome activity. Furthermore, PF1801 suppressed FASLG‐induced reactive oxygen species (ROS) accumulation in myotubes, also crucial for the execution of necroptosis, thorough up‐regulating the antioxidant molecules including *Nfe2l2*, *Hmox1*, *Gclm*, and *Nqo1*.

**Conclusions:**

GLP‐1R agonist could be a novel therapy for PM that recovers muscle weakness and suppresses muscle inflammation through inhi biting muscle fibre necroptosis.

## Introduction

Polymyositis (PM) is a chronic inflammatory myopathy, the primary symptom of which is proximal muscle weakness that leads to progressive and persistent disability. While glucocorticoids (GC) have been the cornerstone of the treatment for PM to suppress immune‐mediated muscle injury, substantial proportion of patients suffer from GC‐induced myopathy during the treatment, which further deteriorates the muscle weakness. Furthermore, significant disability and muscle weakness persist in a quarter of the patients even after successful immunosuppressive therapy.^1^ Therefore, new therapeutic strategies that recover muscle strength as well as suppress inflammation are awaited.

CD8^+^ cytotoxic T lymphocytes (CTLs) play a crucial role to induce muscle fibre death in PM.^2^ Over the past decades, several types of cell death have been identified, one of which, necroptosis is a form of lytic cell death^3^ that is strictly regulated by RIPK1, RIPK3, and MLKL. The cells undergo necroptosis subsequently release inflammatory molecules including intracellular damage‐associated molecular patterns (DAMPs) and cytokines, which cause tissue inflammation and secondary tissue damage.^4^ We have previously shown that the form of cell death of muscle fibres in PM is FASLG‐mediated necroptosis using human muscle biopsy specimens of PM patients and models of PM *in vitro* and *in vivo*.^5^ We also found that necroptosis contributes to the inflammation of PM through the release of HMGB1, one of the family of DAMPs.^5^

While glucagon‐like peptide‐1 receptor (GLP‐1R) agonists have been developed as an anti‐diabetic therapy to promote insulin secretion, emerging data suggest that they have pleiotropic actions including anti‐inflammatory effects,^6^ suppression of muscle wasting,^7^ and inhibition of cell death.^8^ We hypothesized that GLP‐1R agonists have beneficial effects on PM to recover muscle strength and to suppress muscle inflammation through inhibiting muscle fibre death.

PF1801 is a genetically‐engineered GLP‐1R agonist that consists of 636 amino acid polypeptide comprised of GLP‐1 fused to a physiologically inert repeating polymeric elastin‐like peptide to extend the half‐life and slow the rate of absorption of GLP‐1.^9^, ^10^ Its effectiveness and safety profiles on patients with type 2 diabetes have been acknowledged.^9^

Here, we show that GLP‐1R is expressed on the inflamed muscle fibres in PM and PF1801 ameliorates myositis‐induced muscle weakness, muscle atrophy as well as muscle fibre necroptosis and inflammation using PM models *in vivo* and *in vitro*.

## Methods

### Patients and muscle biopsy

Muscle specimens were obtained using percutaneous conchotome muscle biopsy technique^11^ from the tibialis anterior muscle of 12 untreated adult patients with PM (*n* = 9) or dermatomyositis (DM; *n* = 3) who met the Bohan and Peter criteria^12^ and 2017 European League Against Rheumatism/American College of Rheumatology (EULAR/ACR) classification criteria for adult and juvenile idiopathic inflammatory myopathies^13^ at Department of Rheumatology, Tokyo Medical and Dental University (TMDU) between October 2016 and April 2020. All the patients included in the analysis showed necrotic muscle fibres, which were identified with reduced eosin staining in the cytoplasm but did not have the characteristic feature of immune‐mediated necrotizing myopathy.^14^ The patients who were suspicious of cancer‐associated, viral, or immune check point inhibitor‐associated myositis were excluded. The clinical, serological, and histopathological features of the patients were shown in Supporting Information, *Table* S1. The study protocols were approved by the institutional review board at TMDU (reference ID; M2000‐23235‐04 and M2015‐525‐02) and are in accordance with the ethical standards laid down in the 1964 Declaration of Helsinki and its later amendments. Written informed consent was obtained from all participants.

### Mice

C57BL/6 mice were purchased from Charles River Japan (Kanagawa, Japan). The mice were housed at a standard temperature (21 ± 1°C) and in a light‐controlled environment. All animal experiments were approved by the Institutional Animal Care and Use Committee of TMDU (reference ID; A2021‐067C2) and were performed in accordance with the institutional and national guidelines.

### Induction of C protein‐induced myositis (CIM), a murine model of PM

Female C57BL/6 mice at the age of 8 weeks were immunized intradermally with 200 μg of human C protein fragments emulsified in Complete Freund's Adjuvant (CFA) containing 100 μg of heat‐killed Mycobacterium butyricum (Difco, Franklin Lakes, New Jersey, USA). The emulsified C protein fragments were injected at 4 sites: the bilateral base of the tail and the base of bilateral hind limbs of the mice. The emulsified CFA without C protein was also injected subcutaneously into the base of bilateral forelimbs. At the same time, 250 ng of pertussis toxin (Seikagaku Kogyo, Tokyo, Japan) in 200 μL of 0.02% Triton X‐100 (Sigma, St. Louis, Montana, USA) was intraperitoneally injected (IP).^15^

### Treatments of C protein‐induced myositis with PF1801 and/or prednisolone

For the treatment of CIM with PF1801 (ImmunoForge, Seoul, Korea) and/or prednisolone (Sigma, St. Louis, Montana, USA), PF1801 was dissolved in phosphate buffered salts (PBS) and PF1801 or the control vehicle (PBS) was subcutaneously administered every day from day 0 of CIM to day 14 or from day 7 of CIM, just after the measurement of grip strength, to day 21 for the prophylactic treatment or therapeutic treatment, respectively. Prednisolone (PSL; Sigma‐Aldrich, St. Louis, Montana, USA) was dissolved in 0.5% (*w*/*v*) methylcellulose (Wako, Tokyo, Japan) and 5% (w/v) gamma cyclodextrin (Tokyo Chemical Industry, Tokyo, Japan). 20 mg/kg body weight (BW) of PSL or the control vehicle (0.5% methylcellulose and 5% gamma cyclodextrin) was orogastrically administered every day from day 0 of CIM to day 14 or from day 7 of CIM, just after the measurement of grip strength, to day 21 for the prophylactic treatment or therapeutic treatment, respectively. The mice were randomly allocated to each treatment or control group after the induction of CIM. Each cage housed the mice taking different treatments to minimize potential confounders.

### Cell culture of C2C12‐myotubes and time‐lapse imaging

C2C12 cells were cultured in high‐glucose Dulbecco's modified Eagle's medium (DMEM; Sigma‐Aldrich, St. Louis, Montana, USA) supplemented with 10% fetal bovine serum (FBS), penicillin, and streptomycin. To differentiate the myoblasts to myotubes, the myoblasts were cultured until reaching confluence, and then cultured in DMEM supplemented with 2% horse serum for 4–5 days. The myotubes were treated with recombinant mouse FASLG (R&D Systems, Minneapolis, Minnesota, USA) and its cross‐lining anti‐hemagglutinin (HA) antibodies (R&D Systems, Minneapolis, Minnesota, USA) to induce necroptosis. In some experiments, the myotubes were pretreated with 100 μM of benzyloxycarbonyl‐Val‐Ala‐Asp‐fluoromethylketone (z‐VAD‐fmk, BACHEM, Bubendorf, Switzerland), 100 μM of necrostatin‐1s (Nec1s, Haoyuan ChemExpress Co., Ltd., Shanghai, China), 100 nM of PF1801, 10 μM of Compound C (Abcam, Cambridge, UK), or 1 μM of MG132 (Selleck Chemicals, Houston, Texas, USA) for 8 to 24 hours before the treatment with 10 μg/mL of FASLG and 10 μg/mL anti‐HA antibodies or 25 μM of H_2_O_2_ unless otherwise described. The cells death of the myotubes were assessed with time‐lapse imaging as described previously.^16^ Briefly, C2C12 cells were plated on eight‐well chambered slides (Thermo Fisher Scientific, Waltham, Massachusetts, USA) and labelled with CellTracker™ Green (Invitrogen, Carlsbad, California, USA) according to the manufacturer's instructions, as needed. Hoechst 33342 (Thermo Fisher Scientific, Waltham, Massachusetts, USA) was used to stain the nuclei. Dead cells were visualized with propidium iodide (PI; Invitrogen, Carlsbad, California, USA). The myotubes were treated with CellROX (Invitrogen, Carlsbad, California, USA) reagent 30 min before the treatment with FASLG and cross‐linking antibodies or H_2_O_2_ (Wako, Tokyo, Japan) according to the manufacturer's instructions as needed. Images were acquired with a confocal microscope FV10i‐W (Olympus, Tokyo, Japan) every 30 min for the indicated time, and processed with FV10‐ASW and ImageJ softwares. All of the myotubes in five to eight randomly taken microscopic fields which contain about 10–25 myotubes per field were evaluated.

For other, see supporting information.

### Statistical analyses

In the analysis of necrotic area and histological score, Kruskal–Wallis test followed by Dunn's test for non‐parametric analysis was used. In the analysis of weight of quadriceps and spleen, levels of inflammatory mediators, fluorescence intensity of CellROX® in H_2_O_2_‐treated myotubes, and levels of mRNA, one‐way analysis of variance (ANOVA) test followed by Bonferroni post hoc test was used. In the analysis of grip strength, body weight, and the fluorescence intensity of CellROX® in FASLG‐treated myotubes, two‐way ANOVA test followed by Dunnett's multiple comparison test was used. In time course analysis, the statistical significance was determined using Log‐rank test followed by Holm–Sidak multiple comparisons as needed. For parametric analysis, the equivariance was checked using *F* test. All statistical tests were two‐sided. All statistical analyses were performed using GraphPad Prism V.5, 6, and 8.

## Results

### Glucagon‐like peptide‐1 receptor was expressed in inflamed muscles of polymyositis and a murine model of polymyositis

**Figure 1:**
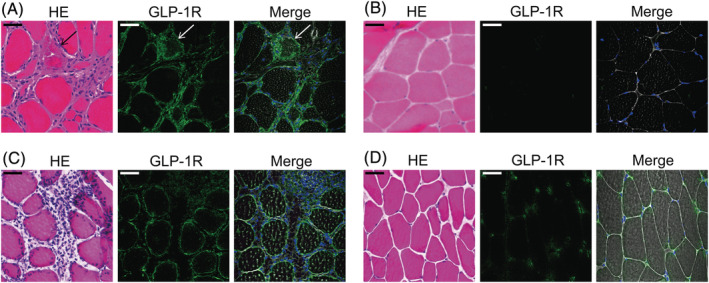
Expression of GLP‐1R in muscle fibres in PM and CIM. (*A, B*) representative images of muscle specimens of PM patients (*n* = 9) of (*A*) inflammatory and (*B*) non‐inflammatory area. Haematoxylin & Eosin (HE) and immunofluorescence staining against GLP‐1R (green). Nuclei were counterstained with DAPI (blue) and merged images of GLP‐1R, DAPI, and phase contrast were shown. Arrow indicates the dying muscle fibre. Scale bar indicates 20 μm. (*C, D*) representative images of muscle specimens of CIM of (*C*) inflammatory and (*D*) non‐inflammatory area. HE and immunofluorescence staining against GLP‐1R (green). Nuclei were counterstained with DAPI (blue) and merged images of GLP‐1R, DAPI, and phase contrast were shown. Scale bar indicates 20 μm.

First, we examined whether GLP‐1R is expressed in the muscle tissue of PM patients (*n* = 9). Immunofluorescence staining revealed that GLP‐1R was expressed on the plasma membrane of muscle fibres, cytoplasm of some of the dying muscle fibres, satellite cells, and some of the inflammatory cells in the inflamed muscles (*Figures* 1A, S1, and S2). Of note, the expression levels on the muscle fibres were high in the area where inflammatory infiltrates were observed compared with those without inflammation (*Figure* 1B). We also examined the muscle specimens of dermatomyositis (DM) patients (*n* = 3), which is another subset of idiopathic inflammatory myopathy, for the expression of GLP‐1R. Consistent with the findings in PM, the expression of GLP‐1R in muscle fibres was confirmed in the plasma membrane of the muscle fibres with inflammatory infiltrates and in the cytoplasm of the dying muscle fibres (*Figure* S1). Next, we examined the expression of GLP‐1R in the muscle of CIM, a murine model of PM.^15^ Consistently, in the inflamed muscles of CIM, GLP‐1R was expressed on the plasma membrane of muscle fibres and on some of the inflammatory cells (*Figure* 1C) while its expression levels on the non‐inflamed muscle fibres were lower (*Figure* 1D).

### Glucagon‐like peptide‐1 receptor agonist suppressed muscle weakness, muscle atrophy, and muscle inflammation in C protein‐induced myositis

**Figure 2:**
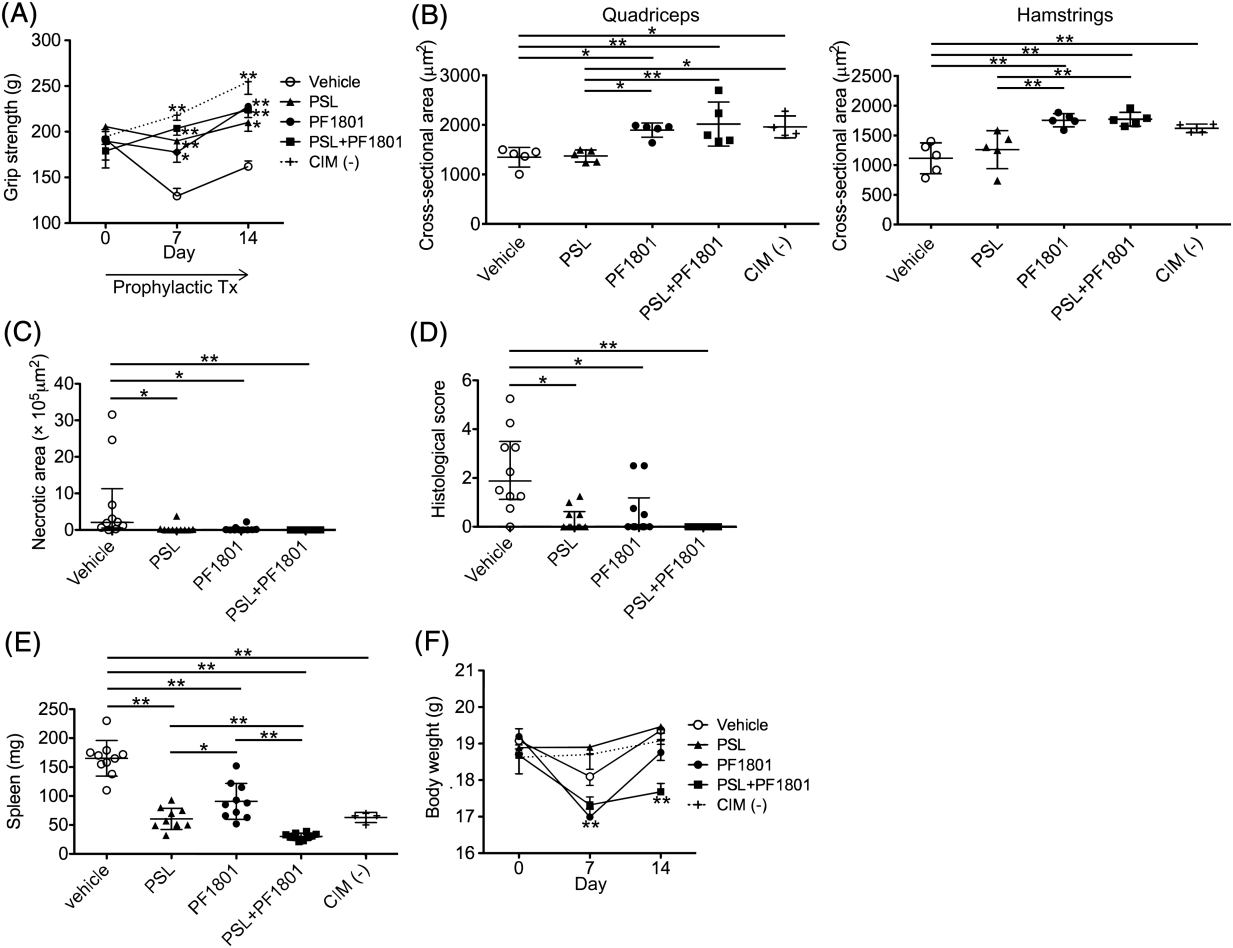
Prophylactic effect of PF1801 on muscle weakness, muscle weight loss, and muscle inflammation in CIM. (*A*) The grip strength of CIM mice treated prophylactically (prophylactic Tx) with PF1801 (*n* = 10), PSL (*n* = 10), combination of PF1801 and PSL (*n* = 10), or vehicle (*n* = 10) and that of non‐CIM mice (*n* = 4). Two‐way ANOVA test, followed by Dunnett's multiple comparison test. *P < 0.05, **P < 0.01. (*B*) The mean cross‐sectional area (CSA) of muscle fibres of rectus femoris (quadriceps) and biceps femoris (hamstrings) of the mice on day 14 of CIM (*n* = 5; vehicle, 5; PSL, 5; PF1801, 5; PSL + PF1801, 4; non‐CIM). Data are presented as mean ± SD. One‐way ANOVA test, followed by Bonferroni post hoc test (all pairs). *P < 0.05, **P < 0.01. (*C*) The area of necrotic muscle fibres on day 14 of CIM. Data are presented as median ± interquartile range. Kruskal–Wallis test, followed by Dunns test. *P < 0.05, **P < 0.01. (*D*) The histological scores of the severity of myositis on day 14 of CIM. Data are presented as median ± interquartile range. Kruskal–Wallis test, followed by Dunn's test. *P < 0.05, **P < 0.01. (*E*) The weight of spleen of the CIM on day 14 of CIM. Data are presented as mean ± SD. One‐way ANOVA test, followed by Bonferroni post hoc test (all pairs). *P < 0.05, **P < 0.01. (*F*) The body weight of the mice. Data are presented as mean ± SD. Two‐way ANOVA test, followed by Dunnetts multiple comparison test. **P < 0.01. (*A–F*) Data represent three independent experiments.

Given that GLP‐1R was expressed on the inflamed or dying muscle fibres of PM/DM and CIM, we sought to determine the effect of GLP‐1R agonist in CIM. First, we administered 5.0 mg/kg/day of PF1801 (ImmunoForge, Seoul, South Korea), a GLP‐1R agonist, on CIM in monotherapy or in combination with prednisolone (PSL) prophylactically from day 0 to day 14 after immunization of C‐protein. CIM resulted in decrease of grip strength compared with that of non‐CIM mice from day 7. Intriguingly, the grip strength was retained in CIM mice treated with PF1801 in monotherapy or in combination with PSL (*Figure* 2A). CIM resulted in decrease of muscle weight on day 14 of CIM compared with that of non‐CIM mice. Although the decrease of the muscle weight in CIM was not ameliorated by the monotherapy with PSL or PF1801, the combination therapy with PSL plus PF1801 suppressed the CIM‐induced muscle weight loss (*Figure* S3A). Histologically, CIM decreased the mean cross‐sectional area (CSA) of the muscle fibres compared with that of non‐CIM mice. The treatment with PF1801 in monotherapy or in combination with PSL retained CSA while the monotherapy with PSL did not (*Figure* 2B). Furthermore, while CIM resulted in shifted CSA size distribution towards a greater proportion of smaller fibres, PF1801 maintained the distribution similar to that in the non‐CIM mice (*Figure* S3B). The necrotic area (*Figure* 2C) and the histological inflammation scores (*Figure* 2D) in the muscles decreased in the mice treated with PF1801 in monotherapy or in combination with PSL (the representative images were shown in *Figure* S3C). We also found the increased spleen weight in CIM compared with that of non‐CIM. The CIM‐induced splenomegaly was suppressed by PF1801 in monotherapy or in combination with PSL. Notably, the additive effect of PSL and PF1801 on the suppression of CIM‐induced splenomegaly was observed (*Figure* 2E). Additionally, the treatment with PF1801 plus PSL resulted in slight decrease in BW from day 7 compared with that of vehicle‐treated mice while the decrease was transient in PF1801 monotherapy (*Figure* 2F).

**Figure 3:**
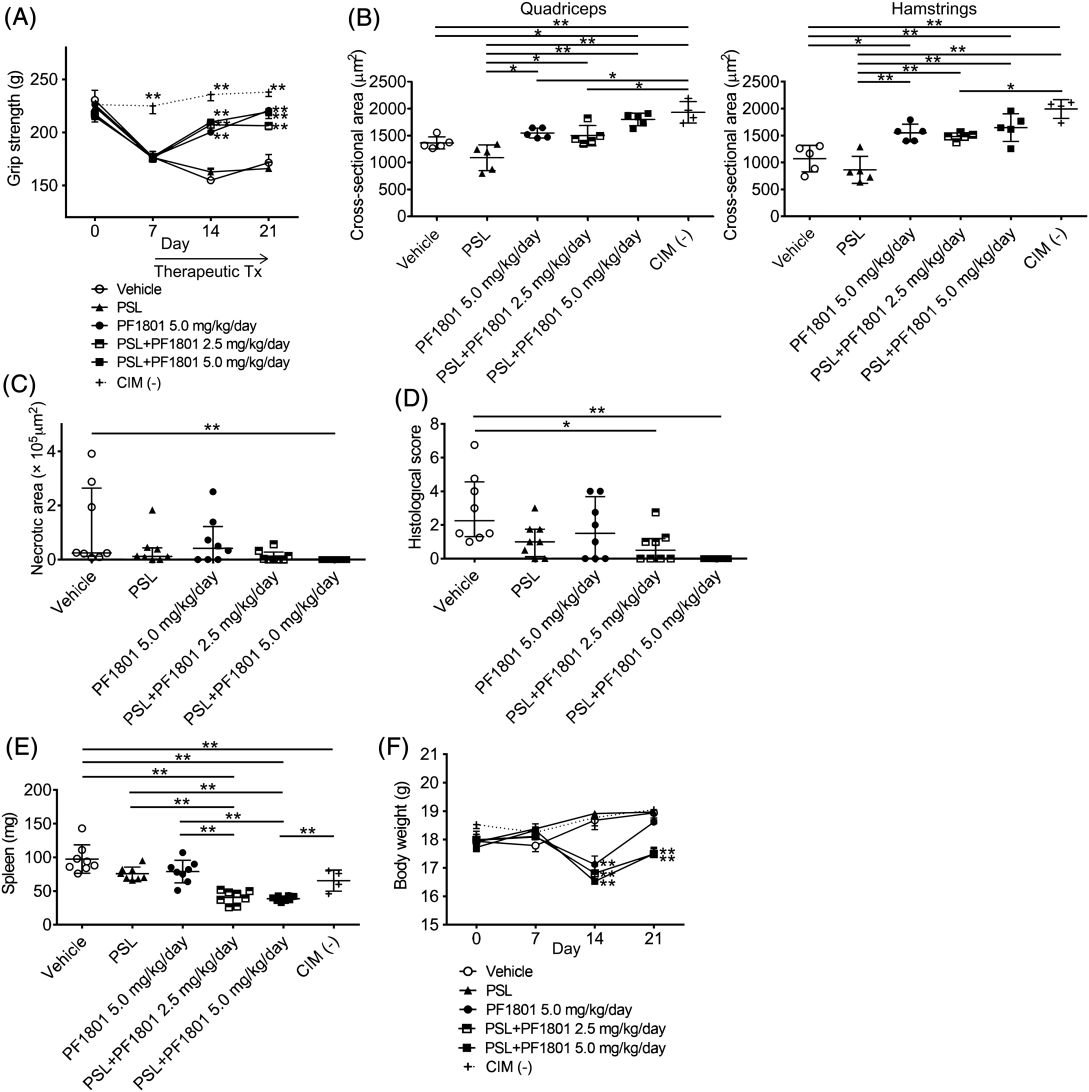
Therapeutic effect of PF1801 on muscle weakness, muscle weight loss, and muscle inflammation in CIM. (*A*) the grip strength of CIM mice treated therapeutically (therapeutic Tx) with PF1801 (5.0 mg/kg/day, *n* = 8), PSL (*n* = 8), combination of PF1801 (2.5 mg/kg/day) and PSL (*n* = 8), combination of PF1801 (5.0 mg/kg/day) and PSL (*n* = 8), or vehicle (*n* = 8) and that of non‐CIM mice (*n* = 4). Two‐way ANOVA test, followed by Dunnetts multiple comparison test. **P < 0.01. (*B*) The mean cross‐sectional area (CSA) of muscle fibres of rectus femoris (quadriceps) and biceps femoris (hamstrings) of the mice on day 21 of CIM (*n* = 5; vehicle, 5; PSL, 5; PF1801 5.0 mg/kg/day, 5; PSL + PF1801 2.5 mg/kg/day, 5; PSL + PF1801 5.0 mg/kg/day, 4; non‐CIM). Data are presented as mean ± SD. One‐way ANOVA test, followed by Bonferroni post hoc test (all pairs). *P < 0.05, **P < 0.01. (*C*) The area of necrotic muscle fibres on day 21 of CIM. Data are presented as median ± interquartile range. Kruskal–Wallis test, followed by Dunns test. **P < 0.01. (*D*) The histological scores of the severity of myositis on day 21 of CIM. Data are presented as median ± interquartile range. Kruskal–Wallis test, followed by Dunns test. *P < 0.05, **P < 0.01. (*E*) The weight of spleen of the CIM on day 21 of CIM. Data are presented as mean ± SD. One‐way ANOVA test, followed by Bonferroni post hoc test (all pairs). **P < 0.01. (*F*) The body weight of the mice. Data are presented as mean ± SD. Two‐way ANOVA test, followed by Dunnett's multiple comparison test. **P < 0.01. (A–F) Data represent two independent experiments.

Next, we examined the therapeutic effect of PF1801 on CIM in monotherapy (5.0 mg/kg/day) or PF1801 (2.5 or 5.0 mg/kg/day) plus PSL administered therapeutically from day 7 to day 21 of CIM. While the monotherapy with PSL did not improve the grip strength, PF1801 in monotherapy or in combination with PSL improved the grip strength of CIM (*Figure* 3A). Muscle weight loss was limited in the mice treated with PF1801 compared with that in PSL or vehicle‐treated mice (*Figure* S3D). PF1801 attenuated the CIM‐induced decrease in CSA and shift in CSA size distribution, the effect of which was remarkable in the combination therapy with 5.0 mg/kg/day of PF1801 plus PSL (*Figures* 3B and S3E). Although neither the monotherapy with PF1801 nor PSL did not ameliorate the necrotic area and inflammation in the muscles, the combination of PF1801 and PSL decreased the necrotic area (*Figure* 3C) as well as the histological inflammation scores (*Figure* 3D), in a PF1801‐dose dependent manner (the representative images were shown in *Figure* S3F). CIM‐induced splenomegaly was suppressed with the combination therapy of PSL and PF1801 and notably, with the additive effect of PF1801 and PSL (*Figure* 3E). While the treatment with PF1801 resulted in slight decrease in BW on day 14 compared with that of vehicle‐treated mice, the difference was reduced on day 21, when especially PF1801 monotherapy and vehicle‐treated mice were comparable (*Figure* 3F).

### Glucagon‐like peptide‐1 receptor agonist suppressed the levels of inflammatory mediators in C protein‐induced myositis

**Figure 4:**
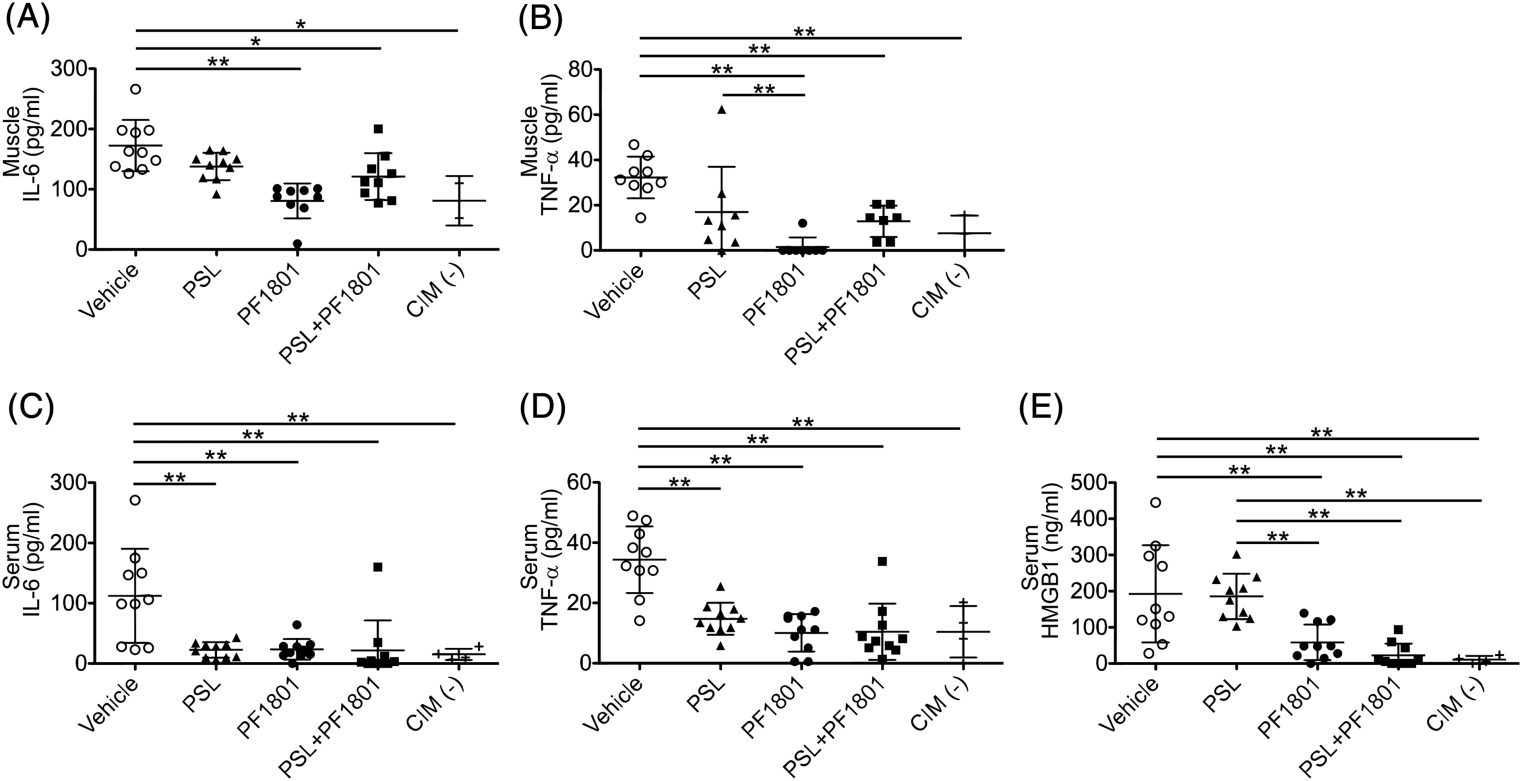
Effect of PF1801 on levels of inflammatory mediators in CIM. (*A, B*) the levels of IL‐6 (*A*) and TNFα (*B*) on day 14 in the muscle homogenates of CIM mice treated prophylactically with PF1801, PSL, combination of PF1801 and PF1801, or the vehicle and that of non‐CIM mice. Bars are mean ± SD. One‐way ANOVA test, followed by Bonferroni post hoc test (all pairs). *P < 0.05, **P < 0.01. (*C–E*) The level of IL‐6 (*C*), TNFα (*D*), and HMGB1 (*E*) on day 14 in the serum of CIM mice. Bars are mean ± SD. One‐way ANOVA test, followed by Bonferroni post hoc test (all pairs). **P < 0.01. (*A–E*) Data represent two independent experiments.

We next studied the effect of PF1801 on the inflammatory mediators in CIM treated prophylactically with PF1801 in monotherapy or in combination with PSL. The levels of TNFα and IL‐6 in the muscle homogenates and TNFα, IL‐6, and HMGB1 in the serum were elevated in CIM mice compared with those in non‐CIM mice. In PF1801‐treated mice, the levels of those inflammatory mediators in the muscle homogenates (*Figure* 4A, B) and in the serum (*Figure* 4C–E) were lower than vehicle‐treated mice, consistent with the histological attenuation of muscle inflammation. Strikingly, the treatment with PF1801 markedly suppressed CIM‐induced increase of HMGB1 in the serum while PSL monotherapy did not (*Figure* 4E).

### PF1801 suppressed FAS‐induced myotube necroptosis and release of inflammatory mediators from myotubes *in vitro*

**Figure 5:**
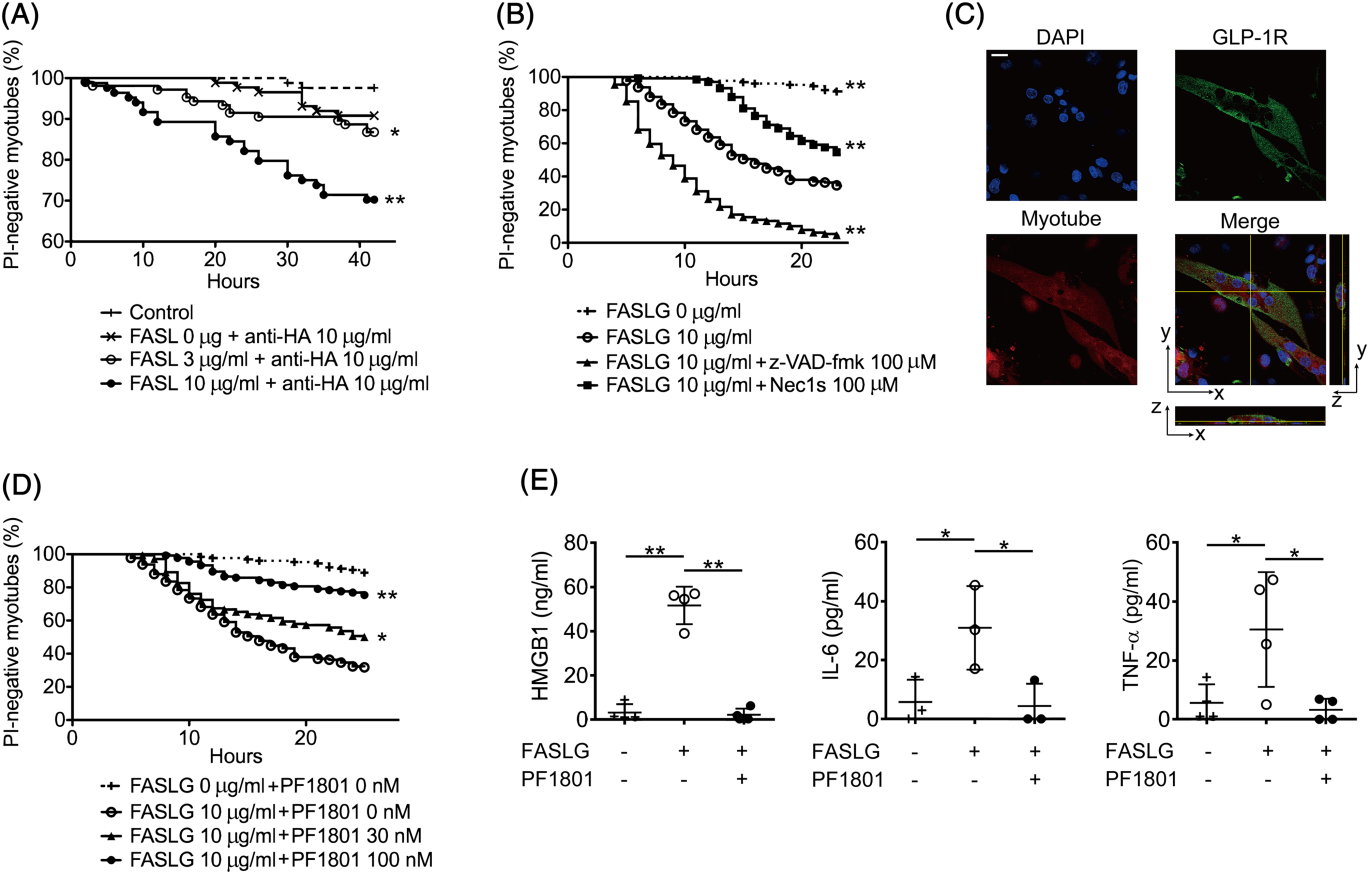
Effect of PF1801 on FASLG‐induced necroptosis in myotubes. (*A*) The viability of FASLG and its cross‐linking anti‐hemagglutinin (HA) antibodies‐treated myotubes (*n* = 84; control, 87; FASLG 0 μg/mL + anti‐HA 10 μg/mL, 106; FASLG 3 μg/mL + anti‐HA 10 μg/mL, 84; FASLG 10 μg/mL + anti‐HA 10 μg/mL). Log‐rank test, followed by Holm–Sidak multiple comparisons. * *P* < 0.05, **P < 0.01. (*B*) The viability of z‐VAD‐fmk‐ or Nec1s‐pretreated myotubes (*n* = 126; FASLG 0 μg/mL, 176; FASLG 10 μg/mL, 129; FASLG 10 μg/mL + z‐VAD‐fmk 100 μM, 132; FASLG 10 μg/mL + Nec1s 100 μM). Log‐rank test, followed by Holm–Sidak multiple comparisons. **P < 0.01. (*C*) The immunofluorescence staining of GLP‐1R (green) in myotubes labelled with CellTracker (red). Nuclei were counterstained with DAPI (blue). Scale bar indicates 10 μm. (*D*) The viability of PF1801‐pretreated myotubes (*n* = 126; FASLG 0 μg/mL + PF1801 0 nM, 176; FASLG 10 μg/mL + PF1801 0 nM, 138; FASLG 10 μg/mL + PF1801 30 nM, 134; FASLG 10 μg/mL + PF1801 100 nM). Log‐rank test, followed by Holm–Sidak multiple comparisons. * *P* < 0.05, **P < 0.01. (*E*) The level of HMGB1, IL‐6, and TNFα in the culture supernatant of the myotubes treated with FASLG for 20 hours. Bars are mean ± SD of quadruple (HMGB1 and TNFα) or triplicate (IL‐6) experiments. One‐way ANOVA test, followed by Bonferroni post hoc test (selected pairs). *P < 0.05, **P < 0.01. (*A, B, and D*) data represent three independent experiments.

We have previously shown that CTL‐induced muscle fibre death in PM is FAS/FASLG‐mediated necroptosis and the inhibition of muscle fibre necroptosis suppressed muscle weakness and inflammation through suppressing the subsequent release of inflammatory mediators including DAMPs and cytokines from dying muscle fibres.^5^ Given the *in vivo* data showing that PF1801 suppressed muscle weakness, necrotic area in the muscles, and inflammation as well as the increase in the levels of inflammatory mediators including HMGB1, we hypothesized that the therapeutic effect of PF1801 on CIM was mediated by inhibiting muscle fibre necroptosis. To investigate the effect of PF1801 in FAS/FASLG‐mediated muscle fibre necroptosis, we employed a modified *in vitro* model of PM we have previously developed.^16^ In this model, C2C12 cell‐derived myotubes were treated with recombinant FASLG and its cross‐linking antibody. Firstly, we confirmed the dose‐dependent cytotoxic effect of FALSG against the myotubes (*Figure* 5A). FASLG‐mediated myotube death was accelerated by a pan‐caspase inhibitor, z‐VAD‐fmk, while suppressed by necrostatin‐1s (Nec1s), a necroptosis inhibitor (*Figure* 5B), indicating that the myotube death in our model was necroptosis rather than apoptosis. In addition, immunofluorescence staining revealed that the myotubes expressed GLP‐1R on their plasma membrane (*Figure* 5C). Next, we tested the effect of PF1801 on myotube necroptosis. PF1801 suppressed the myotube necroptosis in a dose‐dependent manner (*Figure* 5D). Because necroptosis is an inflammatory form of cell death, we then studied the effect of PF1801 on the release of inflammatory mediators from myotubes undergoing necroptosis. While the levels of HMGB1, IL‐6, and TNFα were increased in the supernatants of FASLG‐treated myotubes, the pretreatment with PF1801 prior to FASLG decreased the levels of these inflammatory mediators (*Figure* 5E). Accordingly, PF1801 suppressed muscle weakness and inflammation by inhibiting muscle fibre necroptosis and subsequent release of inflammatory mediators.

### Inhibitory effect of PF1801 on myotube necroptosis was mediated with adenosine monophosphate‐activated protein kinase‐PGAM5 pathway

**Figure 6:**
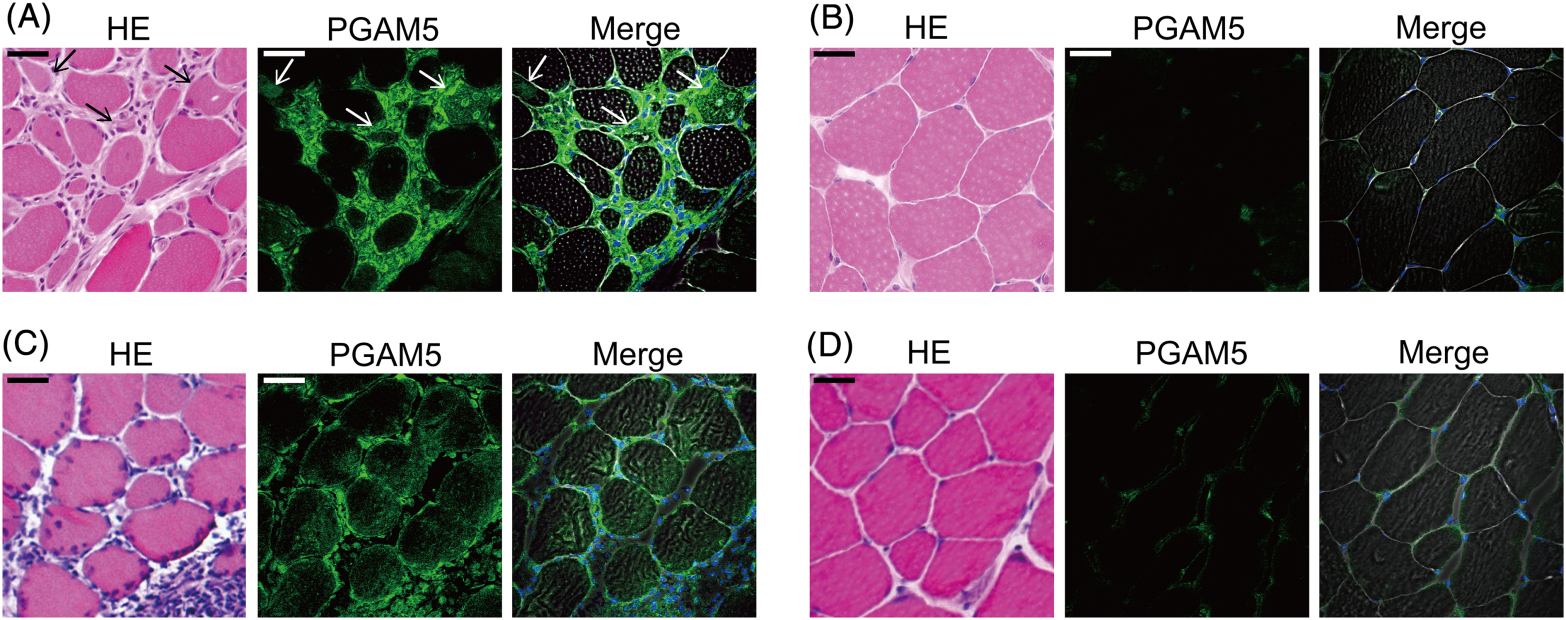
Expression of PGAM5 in muscle fibres in PM and CIM. (*A, B*) representative images of muscle specimens of PM patients (*n* = 9) of (*A*) inflammatory area and (*B*) non‐inflammatory area. HE and immunofluorescence staining against PGAM5 (green). The arrows indicate the dying muscle fibres, which showed reduced eosin staining in the cytoplasm. Nuclei were counterstained with DAPI (blue) and merged images of PGAM5, DAPI and phase contrast were shown. Scale bar indicates 20 μm. (*C*, *D*) Representative images of muscle specimens of CIM of (*C*) inflammatory and (*D*) non‐inflammatory area. HE and immunofluorescence staining against PGAM5 (green). Nuclei were counterstained with DAPI (blue) and merged images of PGAM5, DAPI and phase contrast were shown. Scale bar indicates 20 μm.

**Figure 7:**
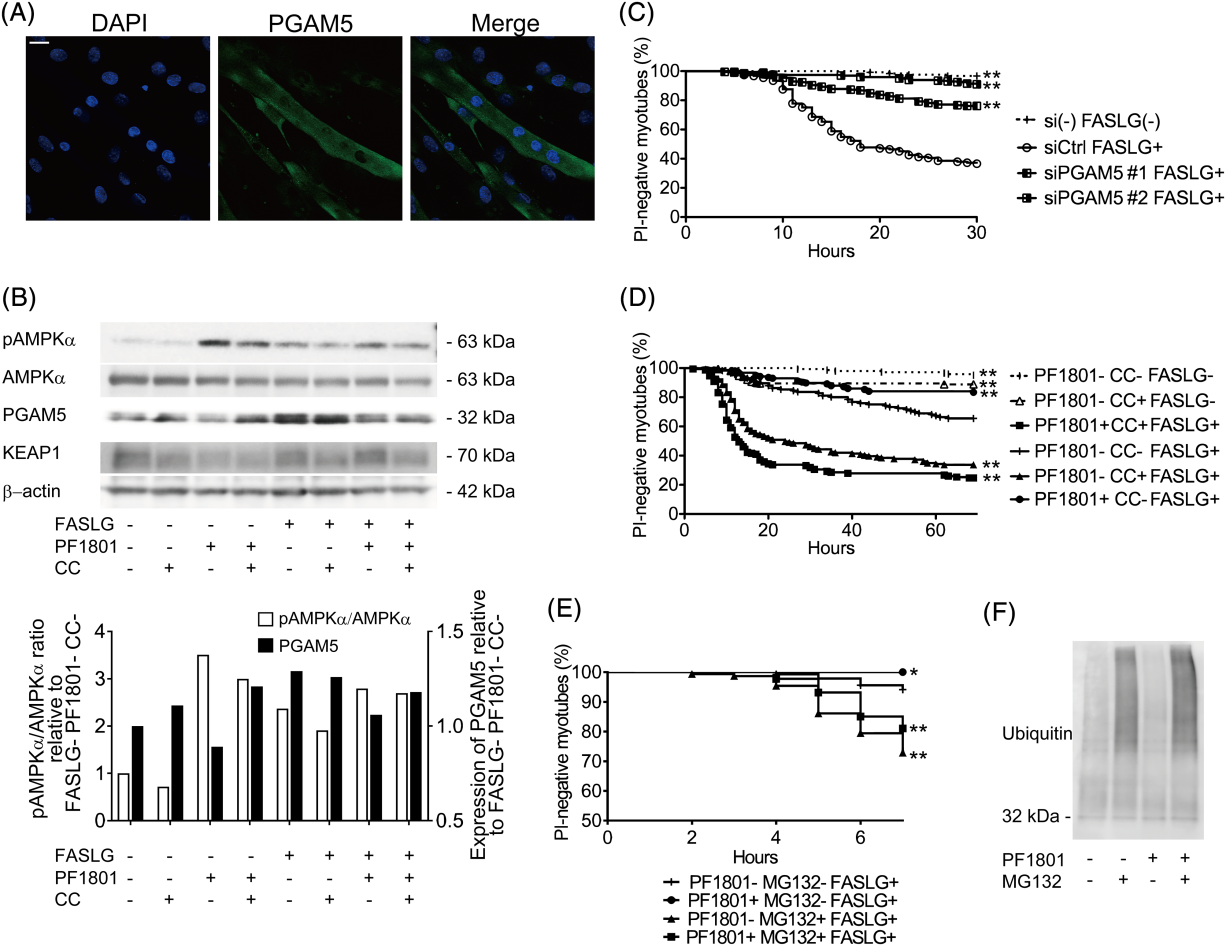
Effect of PF1801 on AMPK‐PGAM5 pathway in FASLG‐mediated myotube necroptosis. (*A*) Immunofluorescence staining against PGAM5 (green) in myotubes. Nuclei were counterstained with DAPI (blue). Scale bar indicates 10 μm. (*B*) The protein expression of pAMPKα, AMPKα, PGAM5, KEAP1, and β‐actin evaluated with western blotting in the myotubes pretreated with PF1801 and/or CC and then treated with FASLG for 12 hours. The relative protein expression in western blot was analysed with ImageJ software. (*C*) The viability of myotubes transfected with scrambled siRNA (siCtrl: *n* = 97) or siRNA specific for *Pgam5* (siPGAM5#1: *n* = 197, siPGAM5#2: *n* = 215) and treated with FASLG and that of myotubes without transfection nor FASLG treatment (siRNA‐FASLG−: *n* = 123). Log‐rank test, followed by Holm–Sidak multiple comparisons. **P < 0.01. (D) The viability of myotubes treated with PF1801, CC, and/or FASLG (*n* = 144; PF1801− CC− FASLG−, 152; PF1801− CC+ FASLG−, 154; PF1801+ CC+ FASLG+, 116; PF1801− CC− FASLG+, 169; PF1801− CC+ FASLG+, 157; PF1801+ CC− FASLG+). Log‐rank test, followed by Holm–Sidak multiple comparisons. **P < 0.01. (*E*) The viability of myotubes pretreated with PF1801 and/or MG132 and then treated with FASLG (*n* = 136; PF1801− MG132− FASLG+, 131; PF1801+ MG132− FASLG+, 151; PF1801− MG132+ FASLG+, 173; PF1801+ MG132+ FASLG+). Log‐rank test, followed by followed by Holm–Sidak multiple comparisons. *P < 0.05, **P < 0.01. (*F*) Immunoblotting analysis of ubiquitin in immunoprecipitated PGAM5 from myotubes treated with PF1801 and/or MG132. (*B–F*) Data represent three independent experiments.

While GLP‐1 has pleiotropic effects, to the best of our knowledge, its necroptosis inhibitory effect has not been reported. Regarding the mechanisms linking GLP‐1R and necroptosis inhibition, we first focused on the previous observations that GLP‐1R agonists stimulated 5′ adenosine monophosphate‐activated protein kinase (AMPK) in muscle^17^ and that AMPK activation inhibited necroptosis through down regulation of PGAM5 in cardiomyocytes.^18^ PGAM5, a protein localized in mitochondria, is essential for mitochondrial fragmentation and functions as a convergent point of necroptosis at least in some cell types.^18^, ^19^ To determine the involvement of PGAM5 in muscle necroptosis in PM, we first evaluated its expression in the muscle specimens of PM/DM patients, CIM, and C2C12‐derived myotubes. PGAM5 was highly expressed in the inflamed or dying muscle fibres of PM/DM patients (*Figures* 6A and S1) while its expression levels were low in uninflamed muscles (*Figures* 6B and S1). Consistent findings were observed in the muscle of CIM (*Figure* 6C, D). Furthermore, PGAM5 was expressed in the myotubes (*Figure* 7A, B), with increased levels by the treatment with FASLG (*Figure* 7B). To discern if PGAM5 is essential for FASLG‐mediated myotube necroptosis, we examined the effect of silencing *Pgam5* with small interfering RNA (siRNA) in the myotubes. Silencing *Pgam5* suppressed FASLG‐mediated myotube necroptosis (*Figure* 7C), indicating that PGAM5 is crucial in myotube necroptosis. Next, to determine the involvement of AMPK in muscle fibre necroptosis, we also examined the expression of phosphorylated AMPKα at Thr172 (pAMPKα), an activated form of AMPKα, in those samples. The expression of pAMPKα was increased in the muscle fibres in inflamed area (*Figures* S1 and S4A) while its expression was low in uninflamed area (*Figures* S1 and S4B) in PM/DM. The findings were consistent in the muscle of CIM (*Figure* S4C, D). Consistent with the findings in PM patients and CIM, the expression of pAMPKα in the myotubes was increased with the treatment with FASLG (*Figure* 7B). To confirm the effect of inactivation of AMPK under the process of necroptosis, we examined the effect of compound C (CC), an AMPK‐kinase inhibitor, on FASLG‐mediated myotube necroptosis *in vitro*. CC enhanced the cytotoxicity of FASLG against the myotubes and cancelled the necroptosis inhibitory effect of PF1801 (*Figure* 7D). Accordingly, AMPK is activated upon necroptosis and inactivation of AMPK further promotes cell death in the presence of necroptosis inducers. In the *in vitro* model, in the absence of FASLG, PF1801 increased the expression of pAMPKα. While FASLG increased the expression of pAMPKα and PGAM5, PF1801 decreased the expression of PGAM5 and the co‐treatment of PF1801 and CC cancelled the suppressive effect of PF1801 on the expression of PGAM5 (*Figure* 7B), suggesting that PF1801 suppressed FASLG‐mediated myotube necroptosis by activating AMPK and down‐regulating PGAM5. PGAM5 is a substrate for redox‐regulated KEAP1‐dependent ubiquitin ligase complex^20^ and AMPK promotes ubiquitination of PGAM5.^18^ We found that PF1801 did not increase the expression of KEAP1 whereas CC decreased its expression (*Figure* 7B). To determine the ubiquitin‐proteasome activity in PGAM5 degradation, we examined the effect of MG132, a proteasome inhibitor, in the *in vitro* model. MG132 promoted FASLG‐induced myotube necroptosis and cancelled the suppressive effect of PF1801 on myotube necroptosis (*Figure* 7E). We also confirmed that immunoprecipitated PGAM5 from the lysates of the myotubes showed a ubiquitin smear when treated with MG132, indicating that proteasome inhibition allowed the accumulation of PGAM5 (*Figure* 7F). We also confirmed in the muscles of CIM mice that the treatment with PF1801 enhanced the expression of pAMPKα and attenuated CIM‐induced elevation in the expression levels in PGAM5 without apparent effect on the expression of KEAP1, which were consistent with the results obtained in the in vitro study (*Figure* S5).

### PF1801 attenuates FASLG‐mediated necroptosis through suppressing accumulation of reactive oxygen species

**Figure 8:**
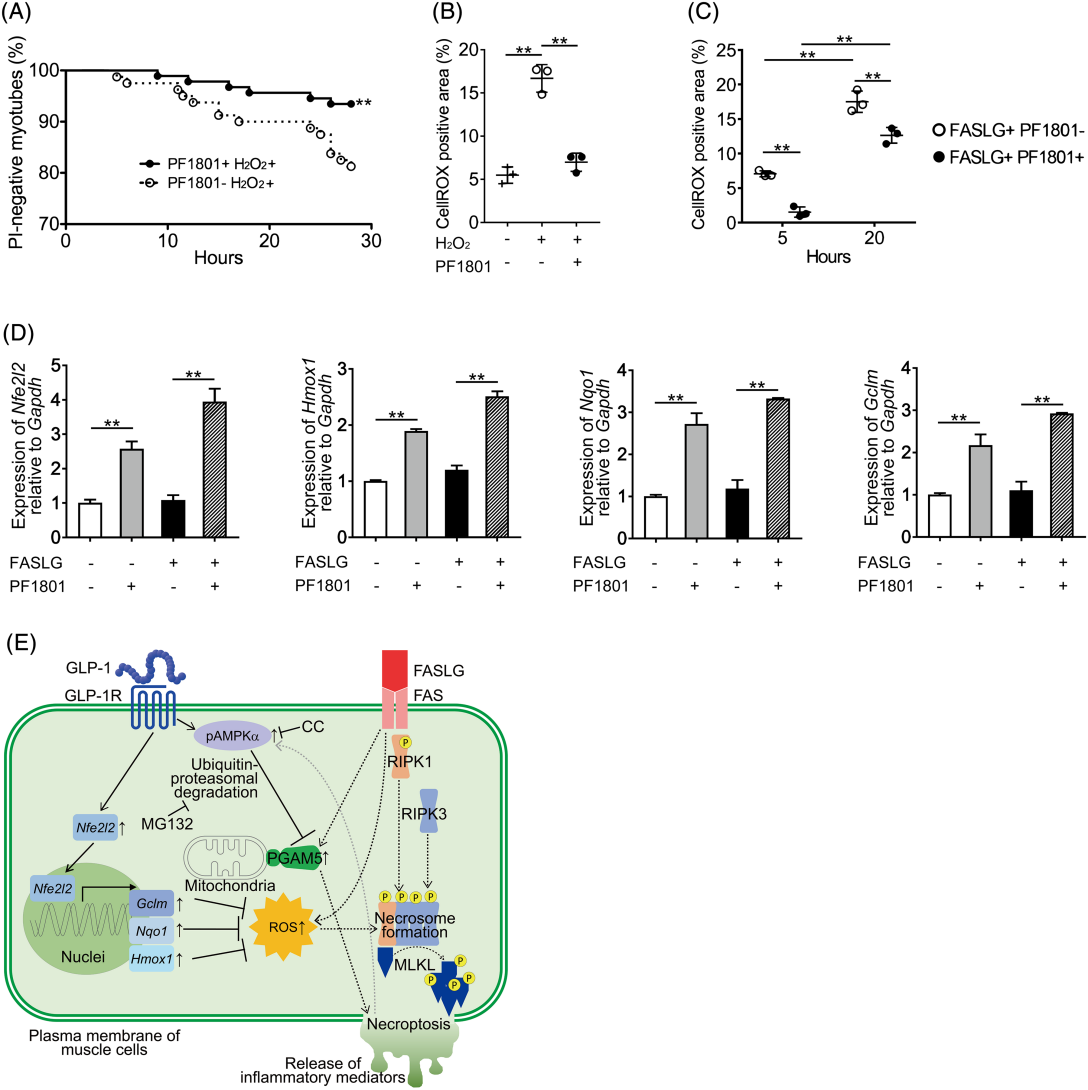
Effect of 1801 on ROS accumulation and expression of antioxidant molecules. (*A*) The viability of the myotubes pretreated with PF1801 and then treated with H_2_O_2_. PF1801− H_2_O_2_+: *n* = 92, PF1801+ H_2_O_2_+: *n* = 80. Log‐rank test. **P < 0.01. (*B*) Fluorescence intensity of CellROX® in the myotubes pretreated or untreated with PF1801 and then treated with H_2_O_2_ for 12 h. Fluorescence intensity of CellROX® was measured in triplicate conditions. Data are presented as mean ± SD. One‐way ANOVA test, followed by Bonferroni post hoc test (all pairs). **P < 0.01. (*C*) Fluorescence intensity of CellROX® in the myotubes pretreated or untreated with PF1801 and then treated with FASLG for indicated hours. Fluorescence intensity of CellROX® was measured in triplicate conditions. Data are presented as mean ± SD. Two‐way ANOVA test. **P < 0.01. (*D*) The mRNA expression of *Nfe2l2*, *Hmox1*, *Nqo1*, and *Gclm* relative to that of *Gapdh* in the myotubes treated with PF1801 and/or FASLG for 4 h. Data are presented as mean ± SD of experiments with duplicate condition. **P < 0.01. (*A–D*) The data represent three independent experiments. (*E*) Schematic representation of the mechanism by which a GLP‐1R agonist suppresses FASLG‐mediated myotube necroptosis. Dashed lines and solid lines indicate the pathways involved in FASLG‐mediated myotube necroptosis and GLP‐1R agonist‐mediated attenuation of cell death, respectively.

Because reactive oxygen species (ROS) promotes necroptosis through enhancing the assembly of necrosome,^21^ which is the core of necroptosis machinery consists of RIPK1 and RIPK3, and GLP‐1 suppresses ROS accumulation by up‐regulating antioxidant proteins in other cell types,^22^ we next hypothesized if PF1801 could also suppress muscle fibre necroptosis through inhibiting ROS accumulation. H_2_O_2_ is a major ROS which induces cell death to various types of cells^18^, ^21^ and we showed that H_2_O_2_ induced cell death (*Figure* 8A) and the increase in ROS levels (*Figure* 8B) to the myotubes. The pretreatment with PF1801 suppressed H_2_O_2_‐mediated myotube death (*Figure* 8A) with decreased ROS levels (*Figure* 8B). The treatment of the myotubes with FASLG also increased their ROS level and PF1801 suppressed the FASLG‐induced increment of ROS (*Figure* 8C). NFE2L2, a transcriptional factor, plays a crucial role in inducing antioxidant genes to decrease the oxidative burden of the cells.^23^ PF1801 increased the levels of *Nfe2l2* and its target antioxidant genes such as *Hmox1*, *Nqo‐1*, and *Gclm* (*Figure* 8D). While KEAP1 is a repressor of NFE2L2,^23^ the expression of KEAP1 in the myotubes was not decreased by the treatment with PF1801 (*Figure* 7B), indicating that PF1801 enhanced the expression of NFE2L2 and its target antioxidant genes without altering the expression of KEAP1.

## Discussion

In this study, we found that GLP‐1R was expressed on the plasma membrane of the inflamed muscle fibres in PM/DM patients and CIM. Furthermore, GLP‐1R agonist ameliorated the muscle weakness, muscle weight loss as well as muscle inflammation through inhibiting muscle fibre necroptosis based on the functional studies with models of PM *in vitro* and *in vivo*. Given that GLP‐1R agonists have been clinically applied as an anti‐diabetic therapy and their detailed information on safety is available, our observations indicated that GLP‐1R agonists could be repositioned as a therapy for PM which not only suppresses muscle inflammation but also improves muscle strength.

Agonistic stimulation of GLP‐1R with PF1801 suppressed myotube necroptosis and subsequent release of inflammatory mediators including HMGB1 through two mechanisms; by down regulating PGAM5 and by suppressing the accumulation of ROS (*Figure* 8I) *in vitro*. We previously revealed that the injured muscle fibres undergo necroptosis, which accompanies with the release of high‐level proinflammatory molecules including HMGB1, leading to further acceleration of muscle inflammation and muscle injury in PM.^5^ Accordingly, suppressing muscle fibre necroptosis and subsequent release of inflammatory mediators is probably one of the key mechanisms by which PF1801 suppressed muscle inflammation in CIM.

We observed rapid improvement of CIM‐induced muscle weakness by PF1801 treatment, suggesting that PF1801 improved muscle fibre functions as well as inhibited the inflammation. Because HMGB1 induces muscle dysfunction through its receptor TLR4,^24^ preventing necroptosis and subsequent HMGB1 release could be a part of the mechanisms under which PF1801 attenuated CIM‐induced muscle weakness. Furthermore, given that ROS induces muscle dysfunction through inducing endoplasmic reticulum (ER) stress,^25^, ^26^ PF1801 could also contribute to the improvement of muscle function through suppressing ROS accumulation.

The therapeutic administration of PF1801 in combination with PSL improved grip strength while the monotherapy with PSL did not, indicating that PF1801 could be effective in both CIM‐ and GC‐induced muscle weakness. GLP‐1 agonists have been reported to ameliorate various murine muscle atrophy models including those induced by GCs, chronic kidney disease, and denervation through suppressing muscle atrophic factors.^7^, ^27^ Moreover, in the GC‐induced muscle atrophy model, GLP‐1R agonist inhibited the translocation of GC receptor into the nuclei.^7^ Furthermore, a clinical study on elderly diabetic patients showed that the patients treated with dipeptidyl peptidase 4 inhibitors had better sarcopenic parameters including skeletal muscle mass and muscle strength than those treated with sulfonylureas and the parameters were correlated with the serum levels of GLP‐1.^28^ Consequently, GLP‐1R agonists could be a potential therapy against muscle weakness caused by diverse conditions as well as PM.

GLP‐1R was expressed in some of the immune cells in the muscle specimens of PM/DM and CIM. Given that GLP‐1R is generally expressed in parenchymal cells rather than immune cells,^29^ most of the reports on the anti‐inflammatory effects of GLP‐1 agonists are considered to be mediated by parenchymal cells such as fibroblasts in arthritis,^6^ dermal cells in psoriasis,^30^ and hepatocytes in nonalcoholic steatohepatitis.^22^ Meanwhile, in several studies on atherosclerosis and obesity, GLP‐1R agonists inhibited the adhesion of monocyte to endothelial cells^31^ and polarized macrophages into anti‐inflammatory subsets.^32^ Therefore, immune cell‐mediated mechanisms might also be responsible for the attenuation of the inflammation in CIM.

Additionally, GLP‐1R is expressed widely in neural tissues including peripheral neuron and GLP‐1R agonists attenuate neural degradation and promote its regeneration in various models of neural damage.^33^ Given that neuromuscular denervation has been proposed to further deteriorate atrophy and muscle weakness in the pathophysiology of inflammatory myopathies,^34^ PF1801 might suppress muscle atrophy or muscle dysfunction in CIM through its neuroprotective effect.

While GLP‐1R agonists have been undergone detailed safety testing with minimal adverse effects, dose‐dependent adverse effects including nausea, diarrhoea, and weight loss are relatively common, which could affect the adherence and persistence with the therapy.^29^ In our *in vivo* experiments, PF1801 resulted in mild and transient weight loss in mice. Therefore, for the clinical applications, the dosage of GLP‐1R agonists needs to be carefully adjusted to balance benefit and side effects, nevertheless this strategy would be a good option for the patients with concomitant diabetes or overweight.

The limitations of this study include relatively small number of the patients analysed for the histopathology, lacking the examination on the effect of the GLP‐1R agonist on immune cells as mentioned earlier, and not evaluating its effect on the extramuscular involvements of inflammatory myopathies including the heart, skin, joint, and lung.^35^ Meanwhile, GLP‐1R agonists have cardioprotective effect^36^ and ameliorated a variety of disease and their models *in vivo* such as psoriasis,^30^, ^37^ arthritis,^6^, ^38^ and pulmonary fibrosis.^39^ Accordingly, GLP‐1R agonists could also be a potential therapy for the extramuscular involvement of inflammatory myopathies.

In conclusion, GLP‐1R agonists would be a novel therapy to restore muscle weakness as well as to suppress muscle inflammation through suppressing muscle fibre necroptosis in PM. Because PF1801 ameliorated PM models through its effects on muscle fibres and is expected to be the therapy which does not directly suppress immune cells or inflammatory mediators, PF1801 is a promising alternative to current immunosuppressive therapies for PM with potentially less infectious complications and more restoration of muscle strength. Clinical trials to test the therapeutic effects of GLP‐1R agonists are awaited in PM, given the excellent therapeutic effects in our preclinical studies.

## Conflict of interest

We declare that F.M. received research funding from AbbVie, Astellas Pharma, Bristol‐Myers Squibb, Chugai Pharmaceutical, Daiichi Sankyo Company, Eisai, Eli Lilly and Company, ImmunoForge, Japan Blood Products Organization, Mitsubishi Tanabe Pharma, Novartis Pharma Japan, Ono Pharmaceutical, Otsuka Pharmaceutical Factory, Pfizer, Sanofi, Takeda Pharmaceutical Company and Teijin, consulting fees from Asahi Kasei Pharma and ImmunoForge, and speaking fees from AbbVie, Asahi Kasei Pharma, Bristol‐Myers Squibb, Chugai Pharmaceutical, Eizai, Eli Lilly and Company, Glaxo Smith Kline, Ono Pharmaceutical, and Pfizer. S.Y. received research funding from Abbvie, Asahi Kasei Pharma, Chugai Pharmaceutical, CSL Behring, Eisai, ImmunoForge, Mitsubishi Tanabe Pharma, and Ono pharmaceutical, consulting fees from ImmunoForge, speaking fees from Abbvie, Asahi Kasei Pharma, Chugai Pharmaceutical, Eisai, Eli Lilly, GlaxoSmithKline, Mitsubishi Tanabe Pharma, Ono pharmaceutical, and Pfizer. M.K. declares that no conflict of interest exists. The authors of this manuscript certify that they comply with the ethical guidelines for authorship and publishing in the *Journal of Cachexia, Sarcopenia and Muscle*.^40^

## Supporting information




**Figure S1.** Expression of GLP‐1R, PGAM5, and pAMPKα in muscle specimens of PM and DM (additional images). Representative images of muscle specimens of eight PM (patient #2–9) and three DM (patient #10–12) donors. Scale bars indicate 20 μm. HE and immunofluorescence staining against GLP‐1, PGAM5, and pAMPKα (green) in the inflammatory area and non‐inflammatory area of the muscles. Arrows indicate the dying muscle fibres, which showed reduced eosin staining in the cytoplasm.
**Figure S2**. Expression of GLP‐1R and dystrophin in muscle fibres in PM. Representative images of muscle specimens of PM patients (*n* = 9) of inflammatory area. HE and immunofluorescence staining against GLP‐1R (green) and dystrophin (red), which localizes in the plasma membrane of muscle cells. Nuclei were counterstained with DAPI (blue) and the merged image of DAPI, GLP‐1R, and dystrophin was shown. Scale bar indicates 20 μm.
**Figure S3**. Effect of PF1801 on muscle weight loss and CSA distribution in CIM and representative histological findings of CIM. (A‐C) The effect of PF1801 on CIM administered prophylactically in monotherapy or in combination with PSL (A) The weight of the quadriceps of the mice on day 14 of CIM mice treated with PF1801 (*n* = 10), PSL (*n* = 10), combination of PF1801 and PSL (*n* = 10), or vehicle (n = 10) and that of non‐CIM mice (*n* = 4). Data are presented as mean ± SD. One‐way ANOVA test, followed by Bonferroni post hoc test (all pairs). **p < 0.01. (B) The frequency distribution of CSA size of muscle fibres in quadriceps and hamstrings (*n* = 5; Vehicle, 5; PSL, 5; PF1801, 5; PSL + PF1801, 4; non‐CIM). Data are presented as mean ± SD. Two‐way ANOVA test, followed by Dunnett's multiple comparison test. *p < 0.05, **p < 0.01. (C) The representative images of HE staining of the muscle of CIM mice. Scale bar indicates 100 μm. (D‐F) The effect of PF1801 on CIM administered therapeutically in monotherapy or in combination with PSL. (D) The wet weight of the quadriceps of the mice on day 21 of CIM mice treated therapeutically (therapeutic Tx) with PF1801 (5.0 mg/kg/day, *n* = 8), PSL (*n* = 8), combination of PF1801 (2.5 mg/kg/day) and PSL (*n* = 8), combination of PF1801 (5.0 mg/kg/day) and PSL (*n* = 8), or vehicle (n = 8) and that of non‐CIM mice (*n* = 4). Data are presented as mean ± SD. One‐way ANOVA test, followed by Bonferroni post hoc test (all pairs). *p < 0.05. (E) The frequency distribution of CSA size of muscle fibres in quadriceps and hamstrings (*n* = 5; Vehicle, 5; PSL, 5; PF1801 5.0 mg/kg/day, 5; PSL + PF1801 2.5 mg/kg/day, 5; PSL + PF1801 5.0 mg/kg/day, 4; non‐CIM). Data are presented as mean ± SD. Two‐way ANOVA test, followed by Dunnett's multiple comparison test. *p < 0.05, **p < 0.01. (F) The representative images of HE staining of the muscle of CIM mice. Scale bar indicates 100 μm.
**Figure S4**. Expression of phosphorylated AMPKα in muscle of PM and CIM. (A, B) Representative images of muscle specimens of PM patients (*n* = 9) of (A) inflammatory area and (B) non‐inflammatory area. (A) HE and immunofluorescence staining against phosphorylated AMPKα (pAMPKα; green). The arrows indicate the dying muscle fibres, which showed reduced eosin staining in the cytoplasm. Nuclei were counterstained with DAPI (blue). Scale bar indicates 20 μm. (C, D) Representative images of muscle specimens of CIM of (C) inflammatory and (D) non‐inflammatory area. HE and immunofluorescence staining against PGAM5 (green). Nuclei were counterstained with DAPI (blue). Scale bar indicates 20 μm.
**Figure S5**. Expression of phosphorylated AMPKα, PGAM5, and KEAP1 in muscle of CIM treated with PF1801. Representative images of muscle specimens of CIM mice on day 14 treated prophylactically with vehicle (*n* = 3) or PF1801 (n = 3) and that of non‐CIM mice (n = 3). HE and immunofluorescence staining against phosphorylated AMPKα (pAMPKα), PGAM5, and KEAP1 (green) in the area where inflammatory infiltrates were observed (inflammatory) or not observed (noninflammatory) were shown. The fluorescence intensity in immunofluorescence staining relative to non‐CIM muscle was analysed with ImageJ software. Data are presented as mean ± SD. One‐way ANOVA test, followed by Bonferroni post hoc test (all pairs). *p < 0.05, **p < 0.01.
**Table S1**. The clinical, serological, and histopathological features of the patients. Bohan and Peter, Bohan and Peter criteria; PM, polymyositis; DM, dermatomyositis; 2017 EULAR/ACR, 2017 European League Against Rheumatism/American College of Rheumatology (EULAR/ACR) classification criteria for adult and juvenile idiopathic inflammatory myopathies; CADM, clinically amyopathic DM; MMT, manual muscle testing; IP, interstitial pneumonia; CK, creatinine kinase (reference interval, male: 62–287 U/L; female: 45–163 U/L); ANA, antinuclear antibodies; MSA, myositis specific antibodies; ARS, anti‐aminoacyl tRNA synthetase antibodies; Jo‐1, anti‐Jo‐1 antibodies; TIF1γ, anti‐transcription intermediary factor 1‐gamma antibodies; SS‐A, anti–Sjögren's‐syndrome‐related antigen A autoantibodies; SS‐B, anti–Sjögren's‐syndrome‐related antigen B autoantibodies; RNP, anti‐ribonucleoprotein antibodies; AMA2, anti‐mitochondrial M2 antibodies; ACA, anti‐centromere antibodies; EMG, electromyography; NA, not analysed; MAC, membrane attack complex. The characters in the EMG findings indicate as follows; (a) short, small, low‐amplitude polyphasic motor unit potentials, (b) fibrillation potentials at rest, and (c) bizarre high‐frequency repetitive discharges.
