# Supplementary material for: Amelioration of inflammatory myopathies by glucagon‐like peptide‐1 receptor agonist via suppressing muscle fibre necroptosis
Source: J Cachexia Sarcopenia Muscle. 2022 Jun 30;13(4):2118–31. doi: 10.1002/jcsm.13025 (PMC9397554; doi:10.1002/jcsm.13025)
Supplement: Supplementary file 1 — Figure S1. Expression of GLP‐1R, PGAM5, and pAMPKα in muscle specimens of PM and DM (additional images). Representative images of muscle specimens of eight PM (patient #2–9) and three DM (patient #10–12) donors. Scale bars indicate 20 μm. HE and immunofluorescence staining against GLP‐1, PGAM5, and pAMPKα (green) in the inflammatory area and non‐inflammatory area of the muscles. Arrows indicate the dying muscle fibres, which showed reduced eosin staining in the cytoplasm. Figure S2. Expression of GLP‐1R and dystrophin in muscle fibres in PM. Representative images of muscle specimens of PM patients (n = 9) of inflammatory area. HE and immunofluorescence staining against GLP‐1R (green) and dystrophin (red), which localizes in the plasma membrane of muscle cells. Nuclei were counterstained with DAPI (blue) and the merged image of DAPI, GLP‐1R, and dystrophin was shown. Scale bar indicates 20 μm. Figure S3. Effect of PF1801 on muscle weight loss and CSA distribution in CIM and representative histological findings of CIM. (A‐C) The effect of PF1801 on CIM administered prophylactically in monotherapy or in combination with PSL (A) The weight of the quadriceps of the mice on day 14 of CIM mice treated with PF1801 (n = 10), PSL (n = 10), combination of PF1801 and PSL (n = 10), or vehicle (n = 10) and that of non‐CIM mice (n = 4). Data are presented as mean ± SD. One‐way ANOVA test, followed by Bonferroni post hoc test (all pairs). **p < 0.01. (B) The frequency distribution of CSA size of muscle fibres in quadriceps and hamstrings (n = 5; Vehicle, 5; PSL, 5; PF1801, 5; PSL + PF1801, 4; non‐CIM). Data are presented as mean ± SD. Two‐way ANOVA test, followed by Dunnett's multiple comparison test. *p < 0.05, **p < 0.01. (C) The representative images of HE staining of the muscle of CIM mice. Scale bar indicates 100 μm. (D‐F) The effect of PF1801 on CIM administered therapeutically in monotherapy or in combination with PSL. (D) The wet weight of the quadriceps of the mice on day [file JCSM-13-2118-s001.pdf]

Supplementary Materials

Supplemental Materials and Methods

Antibodies and reagents

The following antibodies and reagents were used for immunofluorescence staining: anti-GLP-1R (419208, R&D Systems, Minneapolis, Minnesota, USA), anti-GLP-1R (EPR21819, Abcam, Cambridge, UK), anti-dystrophin (rabbit polyclonal, Abcam, Cambridge, UK), anti-PGAM5 (rabbit polyclonal, Abcam, Cambridge, UK), anti-AMPKα (phospho Thr172, D4D6D, Cell Signaling Technology, Danvers, Massachusetts, USA), anti-KEAP1 (1B4, Abcam, Cambridge, UK), polyclonal rabbit IgG (Abcam, Cambridge, UK), monoclonal rabbit IgG (EPR25A, Abcam, Cambridge, UK), anti-rabbit IgG-Alexa Fluor 647 (Thermo Fisher Scientific, Waltham, Massachusetts, USA), anti-mouse IgG-Alexa Fluor 647 (Thermo Fisher Scientific, Waltham, Massachusetts, USA), anti-rabbit IgG-Alexa Fluor 594 (Thermo Fisher Scientific, Waltham, Massachusetts, USA) antibodies, and Fluoromount-G Mounting Medium with DAPI (Thermo Fisher Scientific, Waltham, Massachusetts, USA). For the time-lapse imaging, the following reagents were used: CellTracker^{TM} Green (Invitrogen, Carlsbad, California, USA), CellROX reagent (Invitrogen, Carlsbad, California, USA), benzyloxycarbonyl-Val-Ala-Asp-fluoromethylketone (z-VAD-fmk, BACHEM, Bubendorf, Switzerland), necrostatin-1s (Nec1s, Haoyuan ChemExpress Co., Ltd., Shanghai, China), PF1801 (Immunoforge, Seoul, Korea), Compound C (Abcam, Cambridge, UK), MG132 (Selleck Chemicals, Houston, Texas, USA), recombinant mouse FASLG (R&D Systems, Minneapolis, Minnesota, USA), anti-HA antibodies (R&D Systems, Minneapolis, Minnesota, USA), H_2O_2 (Wako, Tokyo, Japan), Hoechst 33342 (Thermo Fisher Scientific, Waltham, Massachusetts, USA), and propidium iodide (PI; Invitrogen, Carlsbad, California, USA).

For the western blotting, the following regents were used: anti-AMPKα (phospho Thr172, D4D6D, Cell Signaling Technology, Danvers, Massachusetts, USA), anti-AMPKα (rabbit polyclonal, Abcam, Cambridge, UK), anti-PGAM5 (rabbit polyclonal, Abcam, Cambridge, UK), anti-multi ubiquitin (FK2, MBL, Tokyo, Japan), anti-KEAP1 (1B4, Abcam, Cambridge, UK), anti-β-actin (AC-15, Sigma-Aldrich, St. Louis, Montana, USA), horseradish peroxidase (HRP)-conjugated goat anti-rabbit IgG, goat anti-mouse IgG antibodies (Cell Signaling Technology, Danvers, Massachusetts, USA), and Clean-Blot IP detection Reagent (Thermo Fisher Scientific, Waltham, Massachusetts, USA).

Manual muscle testing

Total manual muscle testing (MMT) score of 8 muscles was evaluated before the initiation of treatment based on Kendall's 0-10 point scale in neck extensor, deltoid, biceps, gluteus maximus, iliopsoas, quadriceps, wrist extensor, and ankle dorsiflexor muscles with a maximal score of 150. All of the muscles, except neck extensor muscle, were bilaterally evaluated[1,2].

Measurement of serum CK and autoantibodies

Serum samples were obtained from untreated donors of PM and DM patients who had muscle biopsy, and were analyzed in the clinical laboratory for the levels of CK and autoantibodies. The CK levels were measured by a standard enzymatic method. ANA was measured by a fluorescent antibody method. Anti-ARS antibodies, which bind to Jo-1, PL-7, PL-12, EJ or KS, were measured by enzyme-linked immunosorbent assay (ELISA). Anti-Jo-1, anti-SS-A, and anti-SS-B antibodies were measured by ouchterlony method or ELISA. Anti-TIF1γ, anti-Mi-2, anti-melanoma differentiation associated gene 5 (MDA5), anti-RNP, and anti-centromere antibodies were measured by an

enzyme immunoassay. Anti-mitochondria M2 antibodies were measured by a chemiluminescent enzyme immunoassay.

Assessment of Muscle Strength of CIM mice

The muscle strength of the mice was assessed using a grip strength meter for mice (Muromachi, Tokyo, Japan) as described previously[3]. The mice were picked up in a blinded manner and allowed to grasp the grid of the strength meter with four limbs. The tail of the mice was gently pulled backward until its grasp was broken. The peak force was determined as the grip strength. Five consecutive measurements were made within 30 seconds. The average of three measurements excluding the maximum and the minimum values was determined as the muscle strength. The measurements were performed between 1 pm and 3 pm on day 0, 7, and 14 of CIM or on day 0, 7, 14, and 21 of CIM for the prophylactic treatment and therapeutic treatment, respectively.

Histological Evaluation of CIM

The quadriceps and hamstrings of mice were collected for the histological analysis on day 14 or day 21 of CIM for prophylactic experiments or therapeutic experiments, respectively. The hematoxylin and eosin (H&E) stained 10 μm sections of the quadriceps and hamstrings were examined in a blinded manner for the presence of mononuclear cell infiltration and degeneration of the muscle fibers. The severity of myositis was graded histologically on the scales of 1–6, where 1 = involvement of 1 muscle fiber, 2 = involvement of 2–5 muscle fibers, 3 = involvement of 6–15 muscle fibers, 4 = involvement of 16–30 muscle fibers, 5 = involvement of 31–100 fibers, 6 = involvement of >100 muscle fibers[4]. When multiple lesions with the same grade were found, 0.5 was added to the grade. The score of each muscle was evaluated by averaging scores of 2 different sections. The histological scores of the individual mice were calculated by

summing scores of the quadriceps and hamstrings. The necrotic area of the muscle was measured using ImageJ software. The cross-sectional area (CSA) of muscle fibers in rectus femoris and biceps femoris, which exemplify quadriceps and hamstrings, respectively, using ImageJ software.

Immunofluorescence staining

Muscle specimens from human or mice were frozen for histological analysis. Sections were made in 10 μm thickness. The slides were fixed with 4% paraformaldehyde, permeabilized with 0.1% (v/v) Triton X-100 as needed, blocked with 10% goat serum (Dako Cytomation, Glostrup, Denmark) and 0.1 M glycine in PBS, and then incubated with primary antibodies followed by secondary antibodies. Images were obtained with a confocal laser scanning microscope FV10i-DOC (Olympus, Tokyo, Japan) and processed with FV10-ASW and ImageJ softwares. The fluorescence intensity in immunofluorescence staining was analyzed with ImageJ software.

Enzyme-linked immunosorbent assay (ELISA)

The levels of TNFα and IL-6 of the serum and the muscle homogenate of CIM mice which were treated prophylactically with 5.0 mg/kg BW/day of PF1801 in monotherapy or in combination with 20 mg/kg BW/day of PSL and the supernatant of the FASLG-treated myotubes were measured with Quantikine ELISA kit (R&D Systems, Minneapolis, Minnesota, USA) as described in the manufacturer's instructions. The levels of HMGB1 in the serum of CIM mice and the supernatant of the co-culture were measured with ELISA kit (Shino-test, Kanagawa, Japan) as described in the manufacturer's instructions.

siRNA silencing

C2C12 cells were plated at 5×10^3 cells/cm^2 in DMEM containing 10% FBS. After 48

hours, the cells were transfected with siRNAs or scrambled siRNA (Thermo Fisher Scientific, Waltham, Massachusetts, USA) at 50 nM using Lipofectamine RNAiMAX Reagent (Thermo Fisher Scientific, Waltham, Massachusetts, USA) in DMEM containing 2% horse serum. After 24 hours, the culture media was replaced with DMEM containing 2% horse serum to differentiate the transfected cells into $H2K^{b}$OVA-myotubes.

Western blotting

Cells were lysed with sodium dodecyl sulfate (SDS) lysis buffer (62.5 mM Tris-HCl, pH 6.8, 2.1% SDS, 15% glycerol) supplemented with protease inhibitors (cOmplete, mini, Sigma-Aldrich, St. Louis, Montana, USA). The samples were sonicated with Bioruptor UCD-250 (Cosmo Bio, Tokyo, Japan) followed by centrifugation at 4,000 g for 5 minutes. Protein concentration in the supernatants was measured with Pierce 660 nm Protein Assay Kit (Thermo Fisher Scientific, Waltham, Massachusetts, USA). The samples were denatured with sample buffer (25 mM Tris-HCl (pH 6.5), 1% SDS, 5% glycerol, 0.05% Bromophenol Blue) in the presence of 5% (v/v) 2-mercaptoethanol and boiled for 5 min. 10 μg per lane of the total protein was fractionated with 5-20% SDS-PAGE gel (ATTO Corporation, Tokyo, Japan), and then transferred onto a polyvinylidene difluoride membrane using a transfer apparatus (Bio-rad, Hercules, California, USA). After blocking with 5% bovine serum albumin in TBST (10 mM Tris, pH 8.0, 150 mM NaCl, 0.5% Tween 20) for 30 minutes, the membrane was washed once with TBST and probed with primary antibodies overnight at 4 °C. The membrane was washed for 5 minutes three times with TBST, and incubated with secondary antibodies for an hour. The blots were washed three times with TBST, and developed with the ECL Prime Western Blotting Detection Reagent (GE Healthcare Life Sciences, Buckinghamshire, England). Protein bands were detected with LAS-4000 Imaging

System (Fujifilm, Tokyo, Japan). The relative protein expression in western blot was analyzed with ImageJ software.

Immunoblotting analysis of ubiquitin in immunoprecipitated PGAM5

Cells were lysed with Radio-Immunoprecipitation Assay (RIPA) buffer (50 mM Tris-HCl, pH 7.4, 150 mM NaCl, 0.25% deoxycholic acid, 1% NP-40, 1 mM EDTA). The lysates were immunoprecipitated using anti-PGAM5 antibodies and protein G magnetic beads (Tamagawa Seiki, Nagano, Japan) according to the manufacture's instruction. The immunoprecipitates were denatured with sample buffer (25 mM Tris-HCl (pH 6.5), 1% SDS, 5% glycerol, 0.05% Bromophenol Blue) in the presence of 5% (v/v) 2-mercaptoethanol and boiled for 5 minutes. The samples were fractionated with 5-20% SDS-PAGE gel (ATTO Corporation, Tokyo, Japan) and transferred onto a polyvinylidene difluoride membrane. After the blocking and the incubation with primary antibodies according to western blotting, the membrane was incubated with Clean-Blot IP detection Reagent (Thermo Fisher Scientific, Waltham, Massachusetts, USA) for an hour. The blots were developed with the ECL Prime Western Blotting Detection Reagent.

RNA extraction and quantitative real-time PCR

Total RNA was extracted using RNeasy Mini kit (Qiagen, Hilden, Germany). The concentration and purity of RNA were determined with nanodrop (Thermo Fisher Scientific, Waltham, Massachusetts, USA). Complementary DNA was synthesized with QuantiTect Reverse Transcription Kit (Qiagen, Hilden, Germany). Real-time PCR analysis was conducted using QuantiTect SYBR Green RT-PCR Kit (Qiagen, Hilden, Germany) on a LightCycler 96 real-time thermalcycler (Roche, Mnnheim, Germany). The following primers were used: *Nfe2l2*, forward 5'-

GTGTTCCTTGTTCCCAAAAGC-3’ and reverse 5’-CATCACAGTAGGAAGTTTTAGCAG -3’; *Hmox1*, forward 5’-ACAGAGGAACACAAAGACCAG -3’ and reverse 5’-GTGTCTGGGATGAGCTAGTG-3’; *Nqo1*, forward 5’-TCCATTCCAGCTGACAACC-3’ and reverse 5’-CTTACTCCTTTTCCCATCCTCG -3’; *Gclm*, forward 5’-AATCAGCCCCGATTTAGTCAG-3’ and reverse 5’-CGATCCTACAATGAACAGTTTTGC-3’; *Gapdh*, forward 5’-ACCCAGAAGACTGTGGATGG-3’ and reverse 5’-GTCATCATCCTTGGCAGGTT-3’.

Supplementary figures and legends

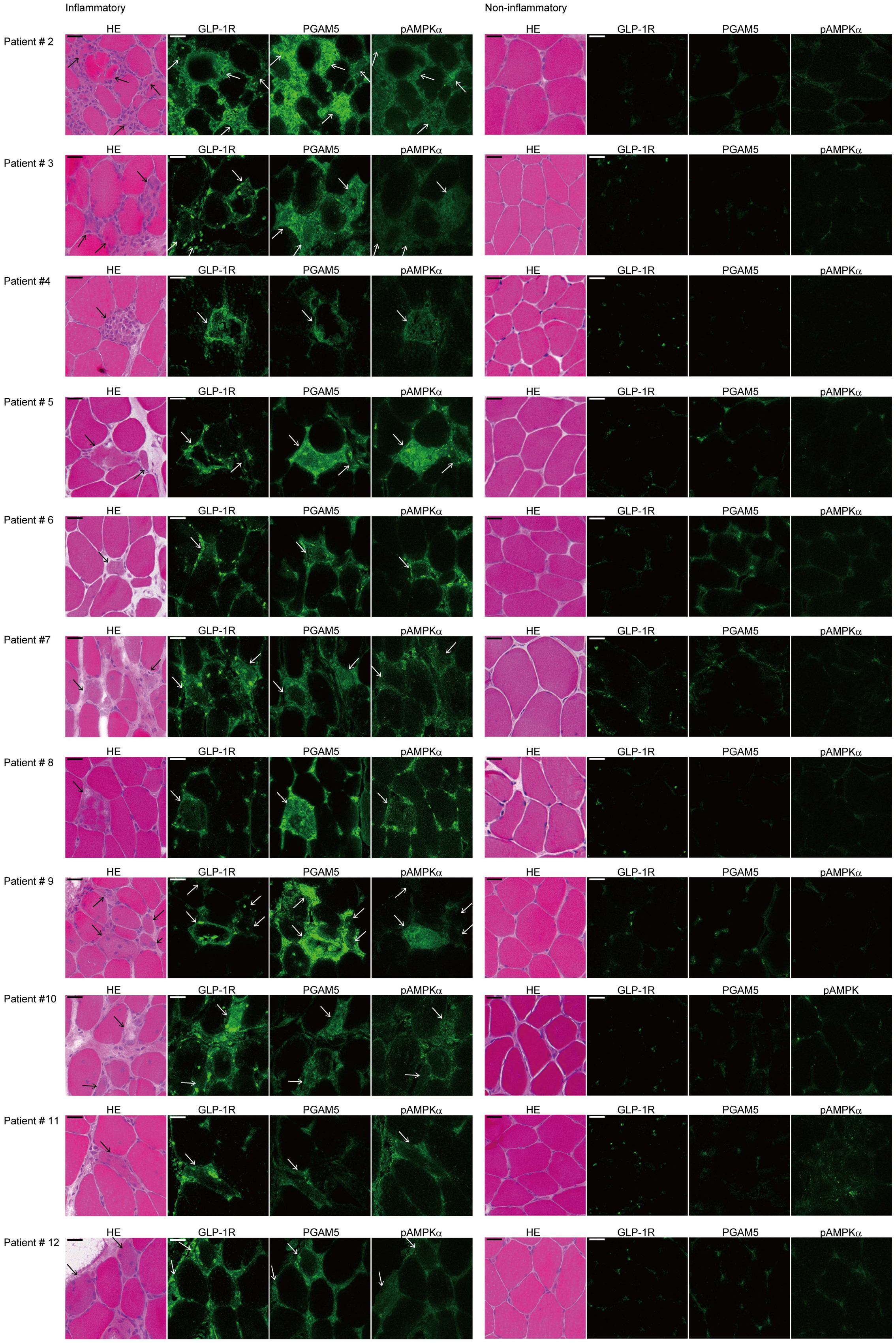

Supplementary Figure 1. Expression of GLP-1R, PGAM5, and pAMPKα in muscle specimens of PM and DM (additional images). Representative images of muscle specimens of eight PM (patient #2-9) and three DM (patient #10-12) donors. Scale bars indicate 20 μm. HE and immunofluorescence staining against GLP-1, PGAM5, and pAMPKα (green) in the inflammatory area and non-inflammatory area of the muscles. Arrows indicate the dying muscle fibers, which showed reduced eosin staining in the cytoplasm.

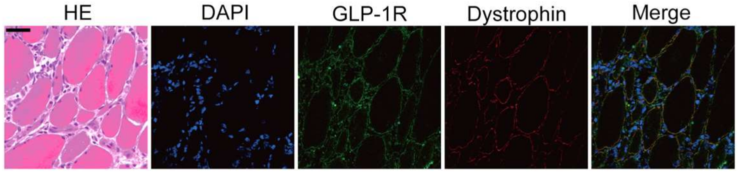

Supplementary Figure 2. Expression of GLP-1R and dystrophin in muscle fibers in PM. Representative images of muscle specimens of PM patients (n = 9) of inflammatory area. HE and immunofluorescence staining against GLP-1R (green) and Dystrophin (red), which localizes in the plasma membrane of muscle cells. Nuclei were counterstained with DAPI (blue) and the merged image of DAPI, GLP-1R, and Dystrophin was shown. Scale bar indicates 20 μm.

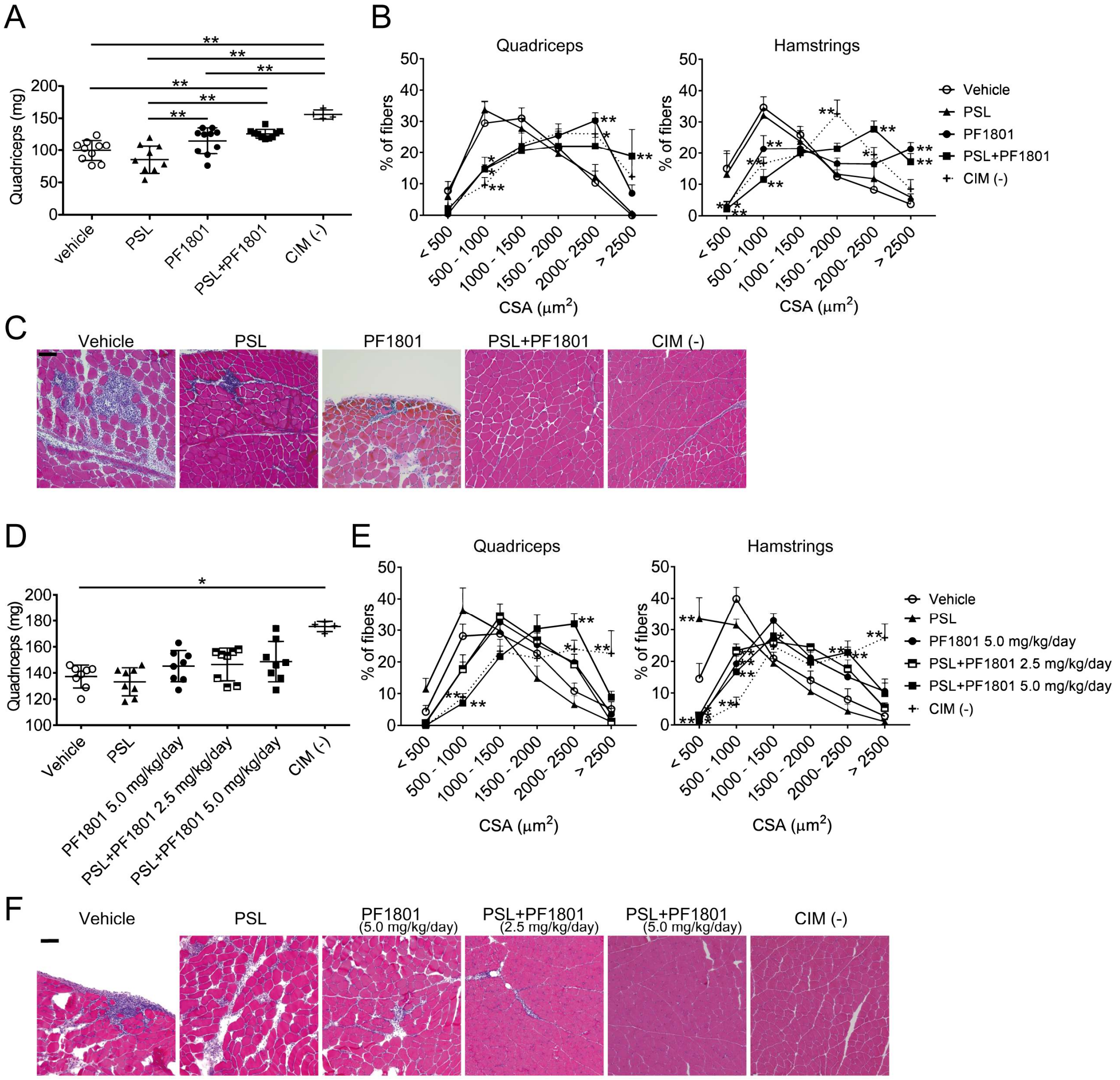

Supplementary Figure 3. Effect of PF1801 on muscle weight loss and CSA distribution in CIM and representative histological findings of CIM. (A-C) The effect of PF1801 on CIM administered prophylactically in monotherapy or in combination with PSL (A) The weight of the quadriceps of the mice on day 14 of CIM mice treated with PF1801 (n = 10), PSL (n = 10), combination of PF1801 and PSL (n = 10), or vehicle (n = 10) and that of non-CIM mice (n = 4). Data are presented as mean ± SD. One-way ANOVA test, followed by Bonferroni post hoc test (all pairs). **$p < 0.01$. (B) The frequency distribution of CSA size of muscle fibers in quadriceps and

hamstrings (n = 5; Vehicle, 5; PSL, 5; PF1801, 5; PSL + PF1801, 4; non-CIM). Data are presented as mean ± SD. Two-way ANOVA test, followed by Dunnett's multiple comparison test. $*p < 0.05$, $**p < 0.01$. (C) The representative images of HE staining of the muscle of CIM mice. Scale bar indicates 100 μm. (D-F) The effect of PF1801 on CIM administered therapeutically in monotherapy or in combination with PSL. (D) The wet weight of the quadriceps of the mice on day 21 of CIM mice treated therapeutically (therapeutic Tx) with PF1801 (5.0 mg/kg/day, n = 8), PSL (n = 8), combination of PF1801 (2.5 mg/kg/day) and PSL (n = 8), combination of PF1801 (5.0 mg/kg/day) and PSL (n = 8), or vehicle (n = 8) and that of non-CIM mice (n = 4). Data are presented as mean ± SD. One-way ANOVA test, followed by Bonferroni post hoc test (all pairs). $*p < 0.05$. (E) The frequency distribution of CSA size of muscle fibers in quadriceps and hamstrings (n = 5; Vehicle, 5; PSL, 5; PF1801 5.0 mg/kg/day, 5; PSL + PF1801 2.5 mg/kg/day, 5; PSL + PF1801 5.0 mg/kg/day, 4; non-CIM). Data are presented as mean ± SD. Two-way ANOVA test, followed by Dunnett's multiple comparison test. $*p < 0.05$, $**p < 0.01$. (F) The representative images of HE staining of the muscle of CIM mice. Scale bar indicates 100 μm.

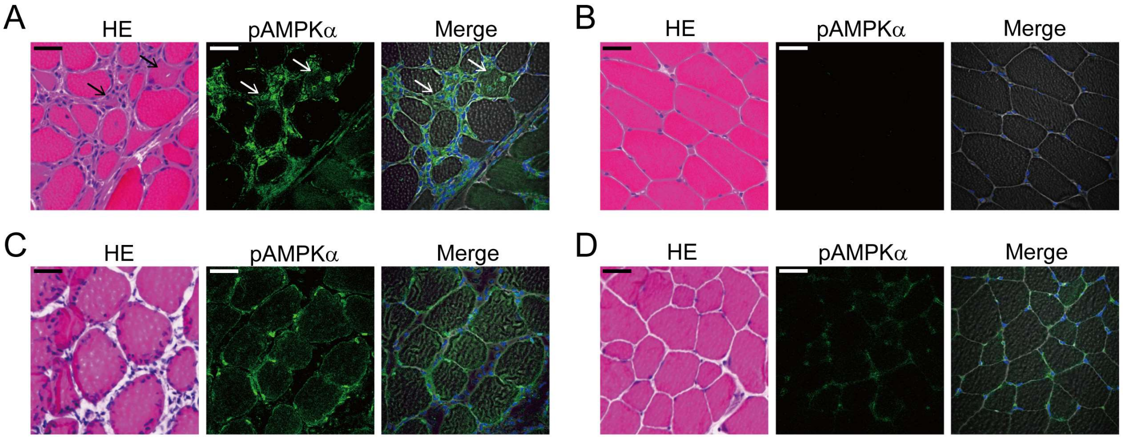

Supplementary Figure 4. Expression of phosphorylated AMPKα in muscle of PM and CIM. (A, B) Representative images of muscle specimens of PM patients (n = 9) of (A) inflammatory area and (B) non-inflammatory area. (A) HE and immunofluorescence staining against phosphorylated AMPKα (pAMPKα; green). The arrows indicate the dying muscle fibers, which showed reduced eosin staining in the cytoplasm. Nuclei were counterstained with DAPI (blue). Scale bar indicates 20 μm. (C, D) Representative images of muscle specimens of CIM of (C) inflammatory and (D) non-inflammatory area. HE and immunofluorescence staining against PGAM5 (green). Nuclei were counterstained with DAPI (blue). Scale bar indicates 20 μm.

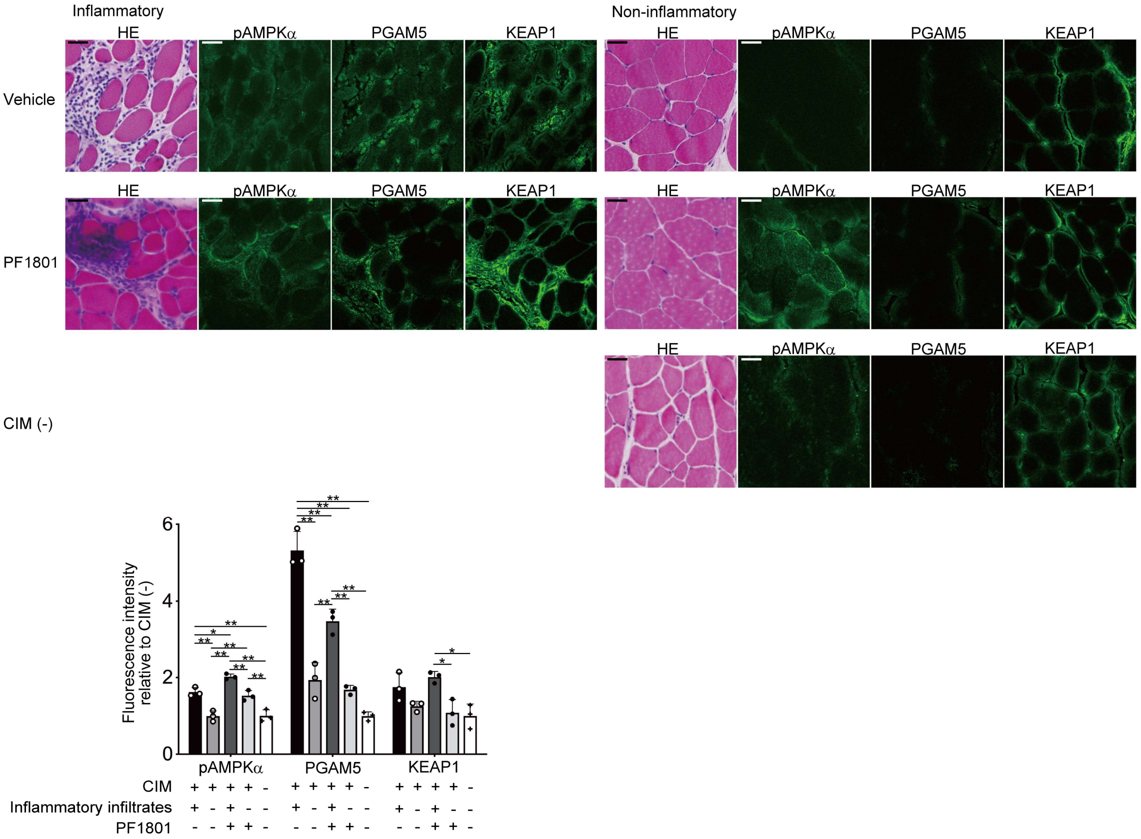

Supplementary Figure 5. Expression of phosphorylated AMPKα, PGAM5, and KEAP1 in muscle of CIM treated with PF1801. Representative images of muscle specimens of CIM mice on day 14 treated prophylactically with vehicle (n = 3) or PF1801 (n = 3) and that of non-CIM mice (n = 3). HE and immunofluorescence staining against phosphorylated AMPKα (pAMPKα), PGAM5, and KEAP1 (green) in the area where inflammatory infiltrates were observed (inflammatory) or not observed (non-inflammatory) were shown. The fluorescence intensity in immunofluorescence staining relative to non-CIM muscle was analyzed with ImageJ software. Data are presented as mean ± SD. One-way ANOVA test, followed by Bonferroni post hoc test (all pairs). *$p < 0.05$, **$p < 0.01$.

Patient's number	Classification criteria		Disease duration	MMT of 8 muscles	Extra-skeletal muscular involvement		
	Bohan and Peter	2017 EULAR/ACR	(months)	(% of max)	Arthralgia	IP	Other
1	Definite PM	Definite PM	8	83.3	+	-	
2	Definite PM	Definite PM	12	84.0	+	+	
3	Definite PM	Definite PM	2	97.3	+	+	Myocarditis
4	Definite PM	Probable PM	6	84.6	+	-	
5	Probable PM	Probable PM	3	100.0	+	+	Fever
6	Probable PM	Definite PM	6	96.0	-	-	
7	Definite PM	Probable PM	12	96.0	-	-	Myocarditis
8	Probable PM	Definite PM	17	86.0	+	-	Pericarditis, Fever
9	Definite PM	Definite PM	12	80.0	-	+	
10	Definite DM	Definite DM	6	91.3	+	+	
11	Definite DM	Definite DM	1	66.0	+	-	
12	Probable DM	Definite CADM	1	100.0	+	+	

Supplementary Table 1. The clinical, serological, and histopathological features of the patients. M, male; F, female; Bohan and Peter, Bohan and Peter criteria; PM, polymyositis; DM, dermatomyositis; 2017 EULAR/ACR, 2017 European League Against Rheumatism/American College of Rheumatology (EULAR/ACR) classification criteria for adult and juvenile idiopathic inflammatory myopathies; CADM, clinically amyopathic DM; MMT, manual muscle testing; IP, interstitial pneumonia; CK, creatinine kinase (reference interval, male: 62-287 U/L; female: 45-163 U/L); ANA, antinuclear antibodies; MSA, myositis specific antibodies; ARS, anti-aminoacyl tRNA synthetase antibodies; Jo-1, anti-Jo-1 antibodies; TIF1γ, anti-transcription intermediary factor 1-gamma antibodies; SS-A, anti–Sjögren's-syndrome-related antigen A autoantibodies; SS-B, anti–Sjögren's-syndrome-related antigen B autoantibodies; RNP, anti-ribonucleoprotein antibodies; AMA2, anti-mitochondrial M2 antibodies; ACA, anti-centromere antibodies; EMG, electromyography; NA, not analyzed; MAC, membrane attack complex. The characters in the EMG findings indicate as follows; (a) short, small, low-amplitude polyphasic motor unit potentials, (b) fibrillation potentials at rest, and (c) bizarre high-frequency repetitive discharges.

Patient's number	Serum CK	Autoantibodies			EMG findings
	(U/l)	ANA	MSA	Other	
1	596	Speckled ×640		SS-A +	a) +, b) +, c) -
2	5394	Speckled ×160, cytoplasmic ×80	ARS + (Jo-1 -), TIF1γ	SS-A +, SS-B +	a) +, b) +, c) -
3	5378	Speckled ×40	Jo-1 +		a) +, b) +, c) -

4	3550	Homogeneous ×80, speckled ×640			a) +, b) +, c) -
5	1035	Speckled ×5120		RNP +	a) +, b) +, c) -
6	6360	< ×40	Jo-1 +		NA
7	1371	Speckled ×40, cytoplasmic ×40		AMAM2 +	a) +, b) +, c) -
8	224	Homogeneous ×80, speckled ×2560		RNP+, SS-A +, SS-B +	NA
9	1533	Speckled ×5120			a) +, b) +, c) +
10	5408	< ×40	ARS + (Jo-1 -)	SS-A +	a) +, b) +, c) -
11	1879	< ×40			NA
12	2212	Discrete speckled ×1280	Jo-1 +	ACA +	NA

Supplementary Table 1 (continued).

Patient's number	Pathological finding	Infiltrating mononucleated cells
1	Endomysial inflammatory cell infiltrate surrounding and invading non-necrotic muscle cells, ubiquitous MHC class I expression	+++, Mainly $CD8^+$ cells and $CD4^+$ cells and a smaller number of $C68^+$ cells. CD20+ cells were scarce.
2	Endomysial inflammatory cell infiltrate surrounding and invading non-necrotic muscle cells, ubiquitous MHC class I expression	+++, Mainly $CD8^+$ cells and $CD4^+$ cells and a smaller number of $C68^+$ cells. $CD20^+$ cells were scarce.
3	Perivascular and perimysial inflammatory cell infiltrates, perifascicular atrophy and MAC depositions on small blood vessels, MHC-class I expression of perifascicular fibers	++, Mainly $CD4^+$ cells and a smaller number of $CD8^+$ cells. $CD68^+$ cells and $CD20^+$ cells were scarce.
4	Perivascular and perimysial inflammatory cell infiltrates, scattered endomysial $CD8^+$ T cells infiltrate that does not clearly surround or invade muscle fibers,<br>perifascicular atrophy and MAC depositions on small blood vessels,<br>MHC-class I expression of perifascicular fibers	++, Mainly $CD4^+$ cells and a smaller number of $CD8^+$ cells and $CD68^+$ cells. $CD20^+$ cells were scarce.
5	Perivascular and perimysial inflammatory cell infiltrates, perifascicular atrophy and MAC depositions on small blood vessels, MHC-class I expression of perifascicular fibers	++, Mainly $CD8^+$ cells and $CD4^+$ cells and a smaller number of $CD68^+$ cells and $CD20^+$ cells.
6	Perivascular and perimysial inflammatory cell infiltrates, scattered endomysial $CD8^+$ T cells infiltrate that does not clearly surround or invade muscle fibers, MAC depositions on small blood vessels without perifascicular atrophy, MHC-class I expression of perifascicular fibers	+, Mainly $CD4^+$ cells and $CD68^+$ cells. $CD8^+$ cells and $CD20^+$ cells were scarce.
7	Perivascular and perimysial inflammatory cell infiltrates,<br>scattered endomysial $CD8^+$ T cells infiltrate that does not clearly surround or invade muscle fibers, no perifascicular atrophy, MAC depositions nor MHC-class I expression	+, Mainly $CD8^+$ cells and a smaller number of $CD4^+$ cells and $CD68^+$ cells. $CD20^+$ cells were scarce.
8	Perivascular and perimysial inflammatory cell infiltrates,<br>scattered endomysial $CD8^+$ T cells infiltrate that does not clearly surround or invade muscle fibers, MAC deposition on small blood vessels without perifascicular atrophy,<br>ubiquitous MHC-class I expression	++, Mainly $CD4^+$ cells and a smaller number of $CD68^+$ cells, $CD8^+$ cells, and $CD20^+$ cells.
9	Perivascular and perimysial inflammatory cell infiltrates,<br>scattered endomysial $CD8^+$ T cells infiltrate that does not clearly surround or invade muscle fibers, MAC deposition on small blood vessels without perifascicular atrophy,<br>ubiquitous MHC-class I expression	+, Mainly $CD4^+$ cells and a smaller number of $CD8^+$ cells and $CD68^+$ cells. $CD20^+$ cells were scarce.

10	Perivascular and perimysial inflammatory cell infiltrates, perifascicular atrophy and MAC depositionsions on small blood vessels, MHC-class I expression of perifascicular fibers	++, Mainly $CD4^+$ cells and a smaller number of $CD68^+$ cells and $CD8^+$ cells. $CD20^+$ cells were scarce.
11	Perivascular and perimysial inflammatory cell infiltrates, scattered endomysial $CD8^+$ T cells infiltrate that does not clearly surround or invade muscle fibers, MAC deposition on small blood vessels without perifascicular atrophy, MHC-class I expression of perifascicular fibers	+, Mainly $CD4^+$ cells and $CD68^+$ cells and a smaller number of $CD8^+$ cells. $CD20^+$ cells were scarce.
12	Perivascular and perimysial inflammatory cell infiltrates, Scattered endomysial $CD8^+$ T cells infiltrate that does not clearly surround or invade muscle fibers, perifascicular atrophy and MAC depositions on small blood vessels, MHC-class I expression of perifascicular fibers	++, Mainly $CD4^+$ cells and a smaller number of $CD8^+$ cells and $CD68^+$ cells. $CD20^+$ cells were scarce.

Supplementary Table 1 (continued).

References

1. Rider LG, Koziol D, Giannini EH, Jain MS, Smith MR, Whitney-Mahoney K *et al.* Validation of manual muscle testing and a subset of eight muscles for adult and juvenile idiopathic inflammatory myopathies. *Arthritis Care Res (Hoboken)* 2010;**62**:465–72.

2. Kendall F, McCreary E, Provance P. *Muscles: Testing and function.* 4th ed. Williams and Wilkins: Baltimore; 1993.

3. Luca A De. Use of Grip Strength Meter to Assess the Limb Strength of mdx Mice. TREAT-NMD Neuromuscular Network SOP M.2.2_001. Washington, DC; 2008.

4. Kimura N, Hirata S, Miyasaka N, Kawahata K, Kohsaka H. Injury and subsequent regeneration of muscles for activation of local innate immunity to

facilitate the development and relapse of autoimmune myositis in C57BL/6 mice. *Arthritis Rheumatol* 2015;**67**:1107–1116.
